# Bionic breathing-type airway exposure system for inhalation exposure studies

**DOI:** 10.3389/fbioe.2026.1871869

**Published:** 2026-09-01

**Authors:** Chenfeng Hua, Liu Yang, Chaojie Qu, Fuwei Xie

**Affiliations:** 1 Key Laboratory of Tobacco Chemistry, Zhengzhou Tobacco Research Institute of CNTC, Zhengzhou, China; 2 Beijing Life Science Academy, Beijing, China

**Keywords:** 3D printed airway, airway exposure, bionic breathing, dynamic gas exchange, inhalation toxicology

## Abstract

To address limitations in anatomical fidelity and respiratory dynamics of animal-based and static *in vitro* models, a personalized bionic airway exposure system was developed as a physiologically relevant alternative. The platform integrates an anatomically accurate human airway replica (trachea to G3 bronchi, reconstructed from chest CT and 3D printing), millisecond-synchronized dynamic breathing control with five waveforms and oral/nasal/combined inhalation routes, branch-level deposition analysis, independent oral and nasal circuits, CO_2_ exchange, and multi-zone exposure interfaces. Using programmable logic control, the system simulates breathing from normal to pathological states, including smoking-integrated patterns, while enabling gas exchange and site-specific exposure testing. Validation demonstrates that the system recapitulates right-dominant airflow distribution (right main bronchus 51.6% ± 0.1% vs. left 48.4% ± 0.1%) and differential particle deposition between inhalation patterns (large-loop 78.0% ± 1.6% vs. small-loop 61.7% ± 1.3%). BEAS-2B cells exhibited dose-dependent cytotoxicity and cytokine release, while hiPSC-derived lung models showed selective inflammatory responses consistent with human airway pathology. This platform provides an *in vitro* model that mimics human respiratory physiology, supporting inhalation toxicology and drug screening while reducing reliance on animal testing.

## 1 Introduction

The respiratory system serves as the critical interface for gas exchange between the human body and the external environment, functioning not only as the primary site for O_2_/CO_2_ exchange but also as a major portal for environmental pollutants to enter the systemic circulation. As primary target organs continuously exposed to ambient air, the oral cavity, nasal passages, and lungs are particularly vulnerable to various harmful agents. Epidemiological studies indicate that respiratory diseases have emerged as one of the leading global causes of mortality, with approximately 3.9 million annual deaths attributed to chronic respiratory diseases (Cohen et al., 2017; Collaborators, 2020; Al Wachami et al., 2024; Burney et al., 2015), imposing substantial socioeconomic burdens. This public health challenge underscores both the importance of inhalation toxicology research and the critical need for developing experimental models capable of accurately simulating human respiratory exposure, which is essential for scientifically evaluating health risks posed by exogenous inhalants (Clippinger et al., 2018; Bailey and Rhomberg, 2020; Lu Phuong et al., 2018; Naidu et al., 2022; Staal et al., 2024).

Current research in inhalation toxicology primarily employs two approaches: rodent-based *in vivo* experiments and *in vitro* alternative tests. However, numerous studies have demonstrated significant limitations in using animal models, particularly the OECD-recommended rat model, for assessing chemical risks in humans (Mowat et al., 2017; Chamanza and Wright, 2015; Mauderly, 1997). These limitations primarily stem from interspecies differences between rodents and humans across multiple aspects, including breathing patterns, anatomical structures, deposition characteristics upon exposure, and clearance/metabolic mechanisms (Fröhlich, 2024; Stucki et al., 2024; Sarangapani et al., 2002a). These interspecies differences manifest in two critical aspects: (1) divergences in inhaled substance deposition patterns, which are governed by airflow dynamics—a parameter strongly influenced by upper/lower respiratory tract anatomy (including airway geometry, dimensions, *etc.*, (Hofmann et al., 1989)); and (2) disparities in pulmonary clearance mechanisms, involving multiple factors such as tissue volume, cellular distribution, mucus properties, macrophage functionality, and biochemical metabolic processes in the airways (Sarangapani et al., 2002b). These fundamental interspecies differences substantially constrain the reliability of animal-to-human extrapolation.

Given the limitations of animal models in predicting the safety of inhaled substances for humans, recent years have witnessed significant advances in the development of *in vitro* inhalation testing models. Current mainstream alternatives include computer simulation-based predictive models and cell-based *in vitro* testing systems (Sadekar et al., 2025). The former approach develops predictive models by integrating key parameters including physicochemical properties of inhaled substances, pharmacokinetic parameters, and anatomical-physiological characteristics of the respiratory system. However, their predictive accuracy is fundamentally constrained by the reliability and precision of the input parameters (Cichocki and Morris, 2017; Kolanjiyil et al., 2019; Mistry et al., 2024; Kolli et al., 2019; Ganguly et al., 2019; Kolanjiyil and Kleinstreuer, 2019; Dang Khoa et al., 2020; Jalink et al., 2020). The latter, at the cellular model level, has benefited from the application of advanced technologies such as organoids and organ-on-a-chip systems, which better simulate inter-tissue interactions and fluidic microenvironments, thereby significantly enhancing the physiological relevance of *in vitro* models (Kastlmeier et al., 2022; Li et al., 2024; Petpiroon et al., 2023; Guerra-Flórez et al., 2023; Masui et al., 2022; Liu J et al., 2024). In particular, the advancement of air-liquid interface (ALI) culture technology has enabled researchers to culture well-differentiated epithelial cells on permeable supports, with the apical side directly exposed to air and the basal side receiving nutrients—a configuration that more closely mimics the *in vivo* situation (Bannuscher et al., 2022; Lenz et al., 2009). Rothen-Rutishauser et al. (Rothen-Rutishauser et al., 2023) systematically summarized the key elements for constructing such lung models, including the selection of scaffold materials, the combination of key cell types (from monocultures to co-cultures with primary cells, immune cells, and fibroblasts), the optimization of culture conditions, and methods for model validation, providing crucial guidance for the development of physiologically relevant *in vitro* lung models.

However, at the level of exposure devices, current technological solutions have failed to keep pace with advancements in cellular models. Widely used exposure platforms, such as the VITROCELL® Cloud series, have made important contributions to standardizing ALI exposure studies, yet their physical design remains highly simplified (Bannuscher et al., 2022; Lenz et al., 2009). Lenz et al. (2009) proposed and validated the “cloud settling” mechanism, utilizing a vibrating mesh nebulizer to generate a dense cloud of micrometer-sized droplets, achieving rapid and uniform aerosol deposition. Bannuscher et al. (2022) through a multi-laboratory comparison study, validated the reliability of the quartz crystal microbalance (QCM) in the VITROCELL® Cloud12 system as a tool for real-time dosimetry, laying the foundation for standardized operation. Although these systems have made significant strides in addressing the questions of “how aerosols deposit” and “how much deposits,” their core exposure mode remains the delivery of aerosols directly into an empty chamber for gravitational settling. This simplified design suffers from three fundamental limitations that hinder its ability to mimic realistic inhalation exposure processes: (1) The absence of anatomical structure: Current systems completely neglect the transport of aerosols through complex three-dimensional geometries such as the oral/nasal cavities, pharynx, larynx, and bronchial tree. Consequently, they cannot recapitulate region-specific deposition phenomena caused by turbulence and inertial impaction dictated by airway geometry, which are critical determinants of the local dose of inhaled substances. (2) The lack of respiratory dynamics: Cloud-settling systems typically employ a single or limited number of aerosol injections, resulting in the settling of all material within minutes by gravitational force, which is far removed from the intermittent, cyclic “inhalation-breath hold-exhalation” physiology of human breathing and thus cannot simulate the impact of key parameters such as tidal volume, breathing frequency, and inhalation-to-exhalation ratio on aerosol deposition efficiency. (3) The singularity of exposure route: These systems typically feature a single aerosol inlet and cannot distinguish between oral and nasal inhalation—two anatomically and physiologically distinct exposure pathways. Ignoring this distinction leads to miscalculations of deposited dose in the upper airways.

This disconnect between “highly biomimetic cellular models” and “highly simplified physical exposure modes” has become a key bottleneck restricting the extrapolation of *in vitro* data to real-world human risk assessment (Zavala et al., 2018; Wieczorek et al., 2023; Guénette et al., 2022; He et al., 2021; Cao et al., 2021; Ward et al., 2020; Küstner et al., 2024; Lovén et al., 2021). While several biomimetic exposure systems have recently been developed—including CT-based airway models (Lizal et al., 2012), programmable breathing systems (Steiner et al., 2020), and organ-chip integrated smokers (Emma Sarles et al., 2024; Sarles et al., 2024; Benam et al., 2020; Kaiser et al., 2021)—each addresses only a subset of the requirements for physiological fidelity (Chen et al., 2024). No existing platform simultaneously integrates anatomically accurate airway geometry, millisecond-synchronized oral/nasal breathing control, and branch-resolved deposition analysis within a single device.

To address this gap, a bionic breathing-type airway exposure system was developed. The main contributions of this study include: (i) anatomically relevant airway reconstruction—a physiologically faithful model of the 0-third generation bronchi constructed from clinical CT imaging and 3D printing; (ii) precise breathing-exposure temporal coordination—millisecond-level (≤0.1 s) synchronisation between respiratory cycles and aerosol delivery using a programmable logic controller; (iii) independent oral, nasal and combined inhalation routes—dedicated oral-branch and nasal-branch circuits enabling differential analysis of deposited doses across distinct entry routes within the same breathing cycle; (iv) bronchial-generation exposure interfaces for regional dose resolution—cell exposure units connectable to different bronchial generations (G0-G3) for direct measurement of region-specific deposition doses, overcoming the single-interface limitation; and (v) integrated physiological microenvironment—CO_2_ exchange and configurable waveforms to simulate alveolar gas exchange and diverse pathophysiological states. Using this platform, system performance was validated through airflow distribution analysis, aerosol deposition characterization, and biological assays. Cigarette smoke exposure of BEAS-2B cells and hiPSC-derived lung models demonstrated dose-dependent cytotoxicity and cytokine responses, requiring higher nominal doses than static systems—consistent with physiological active inhalation. The platform also faithfully reproduced right-dominant airflow distribution and differential particle deposition between oral and nasal inhalation routes. The results demonstrate that by integrating physiologically relevant anatomical structures with dynamic breathing control, this bionic breathing platform enables the precise simulation of the complete process of aerosol inhalation, regional deposition sites, and deposited dose in an *in vitro* model, providing a highly biomimetic experimental system for studying dynamic aerosol deposition patterns and region-specific toxicity. This system holds significant application value for inhalation toxicology research and health risk assessment of complex mixtures such as cigarette smoke and air pollutants.

## 2 Methods

### 2.1 Tracheobronchial tree construction

CT images of the head and thorax from healthy adult male subjects were acquired and reconstructed. The DICOM data were imported into Mimics software (Materialise Inc., Louvain, Belgium) for 3D reconstruction. An initial 3D model of the tracheobronchial tree was automatically generated by the software, followed by manual correction of minor structural defects introduced during automated segmentation. The airway models were validated against established bronchial classification standards, resulting in anatomically complete 3D digital reconstructions of generations 0-3 (G0-G3) tracheobronchial trees.

For physical model fabrication, a stereolithography (SLA) 3D printer (SLA800, Wuhan Yicheng 3D Technology Co., Ltd., China) and transparent photopolymer resin (YC1000, Wuhan Yicheng 3D Technology Co., Ltd., China) were used with a layer thickness of 100 μm and a vertical resolution of 25 μm. The printed models were manually polished using abrasive sticks (grit size 400→800→1200) to achieve a smooth internal surface.

The printed constructs were systematically annotated according to standard anatomical nomenclature, and the luminal diameters at each bronchial generation (G0-G3) were measured using digital calipers (Mahr 4103012, Germany; ±0.01 mm accuracy). This research protocol was approved by the Institutional Review Board of the Second Affiliated Hospital of Zhengzhou University.

### 2.2 Bionic breathing-type airway exposure system

**FIGURE 1:**
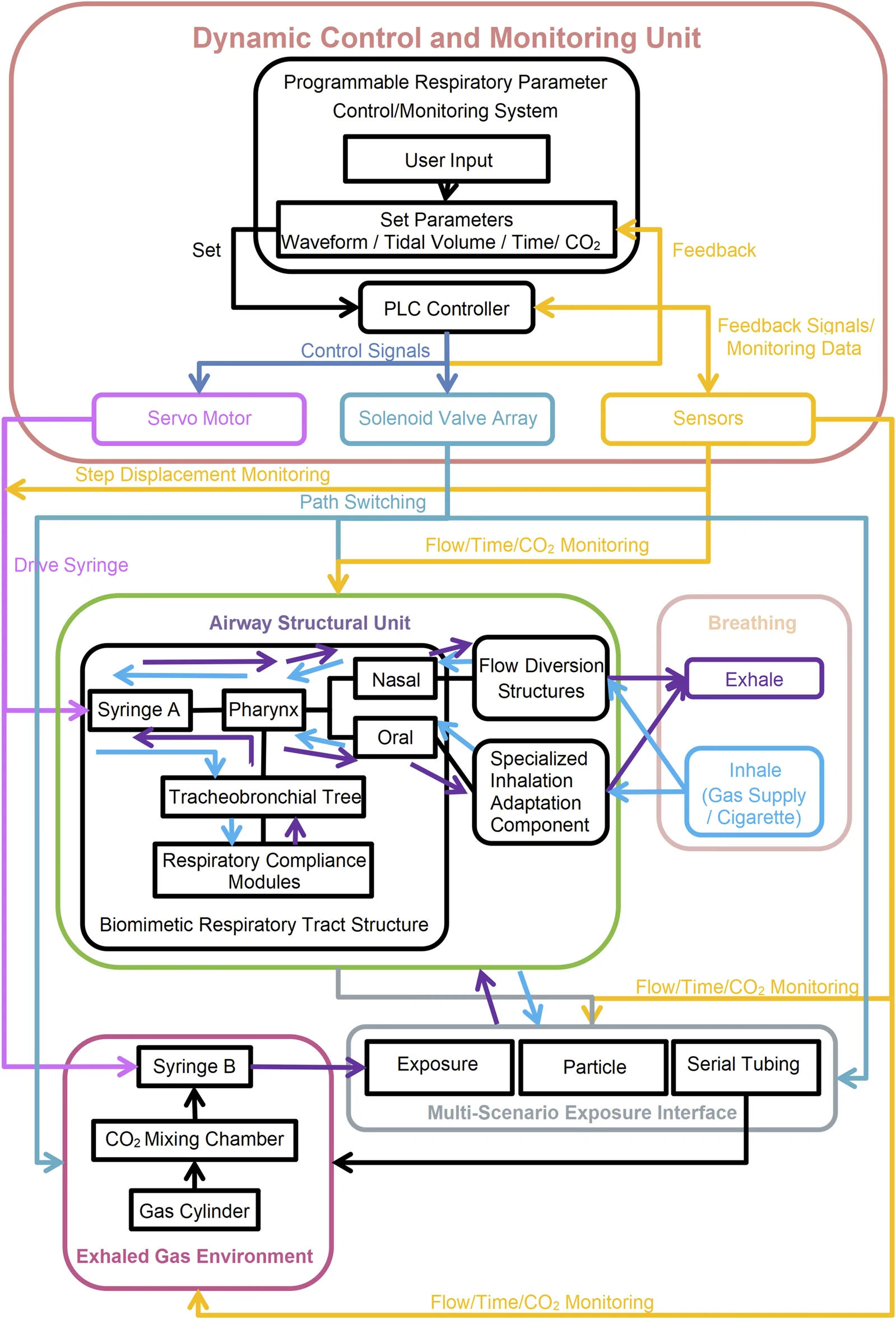
Schematic diagram of the bionic breathing-type airway exposure system. The diagram shows the four core modules (airway structural unit, dynamic control and monitoring unit, exhaled gas environment simulation unit, and multi-scenario exposure interface), the breathing inlet/outlet, and the interconnections.

The bionic breathing-type airway exposure system developed in this study adheres to the design principles of anatomical fidelity and functional completeness. By integrating precisely reconstructed models of human airways from the trachea to third-generation bronchi, and employing programmable logic controller (PLC)-based precision regulation of respiratory parameters, the system achieves a high-fidelity reproduction of real airways and high-precision simulation of the respiratory process in terms of function. The overall architecture and signal flow of the system are illustrated in Figure 1. This schematic integrates four core modules: (1) airway structural unit, (2) dynamic control and monitoring unit, (3) exhaled gas environment simulation unit, and (4) multi-scenario exposure interface. Different colored arrows are used to clearly depict control signals, monitoring signals, and gas flow (inhalation and exhalation). Detailed phase-by-phase valve configurations and airflow paths during the puffing processes, including specific solenoid valve numbers (S1-S8) and descriptions of each phase (I-IV), are provided in Supplementary Material 1.

The airway structural unit includes: (i) biomimetic respiratory tract structure (oral/nasal cavities, pharynx, tracheobronchial tree, and respiratory compliance modules), (ii) specialized inhalation adaptation components, (iii) flow diversion structures, and (iv) syringes A (NSC100X200, Zhejiang Chint Electrics Co., Ltd., China). These components collectively simulate human airway anatomy, oronasal airflow partitioning, and path-specific aerosol distribution under various breathing patterns.

The dynamic control and monitoring unit consists of: a PLC controller (XD5-16T, Xinje Electric Co., Ltd., Wuxi, China), servo motors for syringe actuation (V1000, Suzhou Pengbo Scientific Instrument Co., Ltd., China), electromagnetic valve arrays (WK10/WK04, Woruo Technology, China), a programmable respiratory parameter control/monitoring system (HZV1, Zhengzhou Tobacco Research Institute, China; Chinese Software Copyright Registration Nos. 2025SR1178324, 2025SR0618862, 2025SR0619028, 2025SR0457696), and sensors including limit sensors (GD-sx671-L, Foshan Bangtuosi Technology Co., Ltd., China), flow sensors (MadLab-4C/5H, Beijing Zhongshi Dichuang Technology Development Co., Ltd., China), and gas concentration sensors (XOC1, Suzhou Pengbo Scientific Instrument Co., Ltd., China). The limit sensors are installed at the stepping displacement of the syringes; the flow sensors are directly connected in-line; the gas concentration sensor is embedded in the tubing. This unit precisely regulates and monitors breathing waveforms, tidal volume (0–1000 mL), respiratory rate (including pause and breath-hold durations), CO_2_ concentration, airflow direction/switching, and system operational status, thereby replicating physiological respiratory parameters and simulating respiratory muscle drive, airway resistance, and central control mechanisms. The schematic diagram of the programmable respiratory parameter control/monitoring system interface (as shown in Supplementary Material 2) mainly includes: (i) the action setting interface, (ii) the solenoid valve array control interface, (iii) the continuous action setting interface, and (iv) the operation control/monitoring interface.

Control algorithm: The control algorithm is implemented in the programmable respiratory parameter control/monitoring system (HZV1, Zhengzhou Tobacco Research Institute, China), which consists of four integrated software modules (Software Copyright Registration Nos. 2025SR1178324, 2025SR0618862, 2025SR0619028, 2025SR0457696). This software allows users to program key breathing parameters, including five respiratory waveforms (sine, square, triangular, bell-shaped, and respiratory wave), tidal volume (0–1000 mL), respiratory rate, inhalation/exhalation duration, breath-hold time, and CO_2_ concentration. Based on user-defined settings, the software controls the PLC to execute state transitions (inhalation, breath-hold, exhalation, and inter-cycle pause) via TCP/IP communication, and generates stepping pulses to drive servo motors for precise syringe actuation. Real-time feedback from flow sensors and CO_2_ sensors is displayed on the software interface for monitoring. The software also provides programmable smoking patterns (small-loop, large-loop, and personalized modes), which are assembled from user-defined actions via the “Custom Action” and “Multi-Action Configuration” interfaces, enabling flexible sequence programming without modifying the underlying code. A detailed description of the control logic, including waveform generation formulas, valve sequencing, action assembly procedures, and CO_2_ closed-loop feedback with adjustable deviation values, is provided in Supplementary Material 3.

The exhaled gas environment simulation unit, comprising gas cylinders, a CO_2_ mixing chamber (HZV2, Zhengzhou Tobacco Research Institute, China), and syringe B (NSC100X200, Zhejiang Chint Electrics Co., Ltd., China), collaborates with the control unit to deliver CO_2_ to the exposure chamber according to preset parameters, mimicking alveolar gas exchange. The multi-scenario exposure interface comprises serial tubing (G30, Suzhou Pengbo Scientific Instrument Co., Ltd., China) networks, exposure chambers, and particle collectors. This modular configuration enables flexible integration with mechanical ventilators, aerosol generators, and various exposure units to simulate particulate deposition patterns across different exposure scenarios, including mechanical ventilation and environmental pollutant inhalation. The exposure units consist of commercially available or custom-designed exposure chambers and microfluidic devices capable of housing cell cultures. These units interface with either terminal bronchial branches or oral/nasal positions to enable dynamic, physiologically relevant multi-target exposure simulations for comprehensive toxicological and sensory evaluations. All system parameters were optimized based on smoking and respiratory behavior datasets (as shown in Supplementary Material 4). Detailed design and operation principles of the bionic breathing-type airway exposure system described herein are provided in granted Chinese patents (Invention Patent Nos. ZL202210713943.3 and ZL202210713956.0; Utility Model Patent Nos. ZL202223160280.2 and ZL202223160299.7) and registered software copyrights mentioned above.

#### 2.2.1 Cigarette smoking apparatus and process

Cigarettes were connected to the “oral” inlet of the airway model via an artificial lip and silicone tubing. Negative pressure for drawing smoke was generated by syringe A driven by a servo motor.

#### 2.2.2 Smoking process

During a puff-only sequence (without simultaneous breathing), the PLC opened the solenoid valve at the oral inlet while commanding the servo motor to retract the piston of syringe A, pulling smoke from the cigarette into the system. The puff volume and duration were controlled by the PLC according to preset parameters (e.g., 35 mL/2 s for the small-loop pattern).

During simultaneous smoking and breathing (oral-nasal co-inhalation), the PLC opened both the oral and nasal inlet solenoid valves while retracting syringe A, drawing both cigarette smoke and ambient air (or a supplied gas) into the system. The inhalation volume and duration followed the PLC-preset parameters. The volumetric split between the oral and nasal routes was adjusted by a flow diversion structure connected to the nasal inlet, thereby controlling the amount of smoke drawn through the mouth. Detailed parameters for all smoking patterns are listed in Supplementary Materials 5, 6.

### 2.3 Dynamic inhalation-pulmonary airflow patterns

Three inhalation-breathing composite scenarios were designed to evaluate system performance in terms of accuracy, stability, and personalized adaptability, using cigarette smoking as a representative model. These scenarios were designed based on: (i) vertical deposition gradients (pharyngeal “small-loop” vs. pulmonary “large-loop” patterns) and (ii) population-level respiratory variability. The experimental protocols included: (1) classic small-loop smoking with breathing (7-puff protocol, each cycle: one small-loop puff +3 tidal breaths), (2) classic large-loop smoking with breathing (7-puff protocol, each cycle: one large-loop puff +3 tidal breaths), and (3) personalized smoking with breathing (2-puff protocol repeated for 3 cycles, each cycle: one customized small-loop puff + personalized breathing +1 customized large-loop puff + personalized breathing). System performance was quantitatively assessed through automated operation logging, real-time waveform monitoring, and precision mechanical measurements to capture flow rates, temporal parameters, and waveform characteristics. The terms “small-loop” and “large-loop” smoking patterns refer to the definitions described by (Li et al., 2026): “small-loop” denotes a smoking pattern in which smoke is confined to the oral-pharyngeal cavity without inhalation into the lung, primarily simulating smoke deposition in the oral-pharyngeal region; “large-loop” denotes a smoking pattern in which smoke is swallowed through the larynx and deeply inhaled into the lung. The puffing parameters (puff volume 35 mL, puff duration 2 s) for the classic small-loop and large-loop patterns follow the ISO 3308:2012 standard^52^, which specifies a puff interval of 60 s. In this study, the actual inter-puff intervals resulting from the cycle design of “1 puff +3 tidal breaths” are approximately 35 s and 39 s, which fall within the range of inter-puff intervals reported in the standard (International Organisation for Standardization, 2012; Health Canada, 1999) and in human smoking behavior studies (Zhao and Hua, 2021). The parameters of the personalized smoking patterns (puff volume, various waveforms, arbitrary durations and intervals) are arbitrarily set solely to demonstrate the programmability and flexibility of the system and are not derived from any standard or literature. The key parameters and control parameters of the different breathing patterns are summarized in Table 1 and Supplementary Materials 5, 6.

**TABLE 1:** Key parameters and control parameters of different breathing patterns.

Breathing pattern	Inh/Exh route	Tidal volume (mL)	Waveform	CO_2_ control	Control type
Small-loop puff	Inh: Nasal + oral (air 465, smoke 35); Exh: Nasal	500	Sine/respiratory	None	Open-loop
Tidal breath	Inh: Nasal; Exh: Oral	500 (inh)/510 (exh)	Respiratory	16% CO_2_ 10 mL → 3.57%	Closed-loop
Large-loop puff	Inh: Nasal + oral (air 465, smoke 35); Exh: Nasal	500 (inh)/510 (exh)	Respiratory	16% CO_2_ 10 mL → 3.57%	Closed-loop
Customized small-loop puff	Inh: Oral; Exh: Nasal + oral	300	Sine/square	None	Open-loop
Customized large-loop puff	Inh: Nasal + oral (air 465, smoke 35); Exh: Nasal	500 (inh)/510 (exh)	Respiratory/square/square/respiratory	16% CO_2_ 10 mL → 3.57%	Closed-loop
Personalized breathing	Inh: Nasal + oral; Exh: Oral	800 (inh)/830 (exh)	Bell/sine/triangular/respiratory	30% CO_2_ 30 mL → pathological	Closed-loop

Inh, inhalation; Exh, exhalation. Open-loop means following preset parameters without real-time sensor feedback. Closed-loop means real-time CO_2_ sensor feedback adjusts CO_2_/N_2_ injection valves to maintain target CO_2_ concentration.

### 2.4 Tracheobronchial tree flow distribution analysis

To characterize airflow partitioning across bronchial generations, flow sensors were connected to terminal outlets of each airway generation. Under tidal breathing parameters (tidal volume: 500 mL; breathing waveform: 2 s inspiration; 1 s pause, 2 s expiration; inspiratory-expiratory interval: 1 s), real-time flow rates at all bifurcation levels were recorded via the programmable respiratory parameter control/monitoring system, enabling calculation of generation-specific flow distribution ratios.

### 2.5 Aerosol deposition characterization

Standard-weight research cigarettes (Zhengzhou Tobacco Research Institute of CNTC, Zhengzhou, China) were machine-smoked following the classic small-loop and large-loop smoking with breathing scenarios established in Section 2.3 (7 puffs/cigarette, 4 cigarettes/filter pad). Aerosol particulate matter was quantitatively collected using Cambridge filter pads at both: (i) The inhalation port (M_in_), (ii) The exhalation port (M_ex_). The deposition efficiency was calculated as: Smoke deposition rate (%) = [(M_in_ - M_ex_)/M_in_] × 100.

### 2.6 Pulmonary airway cell exposure assessment

#### 2.6.1 Establishment of airway cell exposure model

BEAS-2B (American Type Culture Collection, USA) cells were seeded at a density of 1 × 10^5^ cells/well in 6-well Transwell plates (Cat. 3450, Corning, USA) and cultured in submerged conditions in BEBM medium (CC3171/CC4175, Lonza, USA) within a HERAcell 240 incubator (Thermo Scientific, Germany) for 24 h. Subsequently, the cells were transferred to exposure chambers (Beijing Huironghe Technology Co., China) adapted for 6-well Transwell inserts, containing fresh medium. Based on the flow distribution data acquired in Section 2.4, the exposure apparatus containing BEAS-2B cells was connected to the RB5 bronchial branches, with real-time aerosol flow monitoring at the inlet. Under the classic large-loop smoking with breathing scenario, cells were exposed to cigarette smoke. The exposure dose was calculated as: Dose (puffs) = n × V_in_/V_total_, where n = total puff number, V_in_ = gas volume entering the exposure chamber, and V_total_ = total tracheal inflow. For exposure units connected to oral or nasal interfaces, the actual delivered dose requires comprehensive adjustment accounting for: (1) specialized puff topography parameters (volume/duration/frequency), (2) exhalation route-specific particle deposition (oral/nasal/oronasal), and (3) real-time aerosol deposition dynamics.

#### 2.6.2 Respiratory influence assessment

Static culture and air-exposed control groups (24 cycles of classic large-loop air breathing, 2 h) were established. Flow monitoring confirmed consistent intake volume (20.53 ± 0.24 mL/cycle) into the exposure chamber. Cell viability was assessed using CCK-8 assay (Cat. CK-04, Dojindo Laboratories, Japan), with the medium pH measured before and after the experiment using a Metrohm 702 SM Titrion automatic potentiometric titrator. O_2_ and CO_2_ concentrations were monitored in real time by the programmable respiratory parameter control/monitoring system to exclude the influence of respiratory dynamics alone on cell viability.

Concentration monitoring and control: The CO_2_ and O_2_ concentrations inside the exposure chamber were monitored in real time by the programmable respiratory parameter control/monitoring system via gas concentration sensors. For CO_2_ concentration, the system compared the measured values with the preset target and automatically adjusted the opening/closing of CO_2_ and N_2_ injection valves as well as the ventilation flow rate to maintain CO_2_ within the target range. O_2_ concentration was indirectly regulated by adjusting the concentration and volume of CO_2_/N_2_ in conjunction with real-time monitoring. Detailed sensor configuration and control logic are provided in Section 2.2.

#### 2.6.3 Cigarette smoke exposure evaluation

Three exposure regimens were established, all completed within 2 h: air control group (24 cycles of classic large-loop air breathing); mixed exposure group (12 cycles of classic large-loop smoking with breathing +12 cycles of classic large-loop air breathing); and full smoke exposure group (24 cycles of classic large-loop smoking with breathing). The corresponding exposure doses for the three regimens were 0 puffs, 3.45 puffs, and 6.90 puffs, respectively. Based on the growth surface area of the 6-well Transwell insert (4.67 cm^2^), the normalized surface area doses (puffs/cm^2^) were calculated as number of puffs/growth surface area (cm^2^), yielding 0 puff/cm^2^, 0.74 puff/cm^2^, and 1.48 puff/cm^2^ for 0, 3.45, and 6.90 puffs, respectively. Immediately following exposure, the following assays were performed: cell viability was assessed using the CCK-8 assay by adding CCK-8 solution per well, incubating at 37 °C for 2 h, and measuring absorbance at 450 nm using a microplate reader (MD Flex3, Molecular Devices, USA); cell culture supernatants were collected, and human IL-6 and IL-8 secretion levels were quantified using ELISA kits (R&D Systems, USA) according to the manufacturer’s instructions.

### 2.7 Exposure assessment of hiPSC-derived lung model and molecular assays

#### 2.7.1 Differentiation of hiPSC-derived lung model

To overcome the limitations of traditional monolayer cell models and investigate cigarette smoke toxicity from a more physiologically relevant perspective, a human induced pluripotent stem cell (hiPSC)-derived lung model was employed in this study. hiPSCs (iCell-DRY0100, Saibai Kang/iCell, China) were seeded into Matrigel-coated 24-well Transwell® inserts (Cat. 3470, Corning, USA) at a density of 4.5 × 10^6^ cells/mL according to an established 27-day differentiation protocol in our laboratory. hiPSCs were maintained in mTeSR™ medium. For differentiation, DMEM/F-12 was used as the basal medium, with sequential addition of specific growth factors and small molecule modulators to direct the cells through definitive endoderm, anterior foregut endoderm, and lung progenitor cells, ultimately generating a mature respiratory epithelial-like lung model. This differentiation protocol was established in our laboratory (patent pending, Chinese Patent Application No. 202511619009.5). Detailed concentrations, factors, and culture conditions are provided in Supplementary Material 7 (Supplementary Tables S3-S6).

Upon completion of differentiation, the model was systematically characterized. Barrier function was first assessed: expression and localization of the tight junction protein ZO-1 were detected by immunofluorescence staining. The immunofluorescence staining procedure was as follows: cells were fixed with 4% paraformaldehyde for 15 min, permeabilized with 0.2% Triton X-100 for 10 min, blocked with 5% BSA for 1 h, and incubated with rabbit anti-ZO-1 polyclonal antibody overnight at 4 °C. The next day, cells were incubated with Alexa Fluor 488-conjugated goat anti-rabbit IgG secondary antibody for 1 h at room temperature in the dark, counterstained with DAPI (1 μg/mL) for 5 min, and observed under a fluorescence microscope for image acquisition. Subsequently, cellular composition was characterized using the same immunofluorescence staining method to detect the following markers: proximal airway epithelial lineage markers (KRT5, ACTUB, CC10, MUC5AC), distal alveolar epithelial lineage markers (PDPN, SP-C, SP-B), and markers for other cell populations (CHGA, SOX2, SOX9, PDGFR-α, CD31, CD68). For details on the reagents related to hiPSC-derived lung model differentiation, see Supplementary Material 7.

#### 2.7.2 Cigarette smoke exposure

Mature hiPSC-derived lung models with comparable transepithelial electrical resistance (TEER) values were selected and exposed to cigarette smoke generated under the “classic large-loop” inhalation mode as described in Section 2.6.3. An air-exposed group was established as the negative control. The gas volume entering the exposure chamber per respiratory cycle was 3.42 mL. Based on the growth surface area of the 24-well Transwell insert (0.33 cm^2^), the surface area normalized dose (puffs/cm^2^) was calculated as number of puffs/growth surface area (cm^2^). Exposure doses were 0 puffs and 1.15 puffs, corresponding to surface area normalized doses of 0 puff/cm^2^ and 3.48 puffs/cm^2^, respectively.

#### 2.7.3 Post-exposure assays

The following assays were performed immediately after exposure:

Epithelial Barrier Function: TEER was measured using an EVOM4 Epithelial Voltohmmeter (World Precision Instruments, USA) before exposure (baseline) and 30 min after exposure. Results were expressed as percentage of control values. Meanwhile, expression and localization of the tight junction protein ZO-1 were detected by immunofluorescence staining as described in Section 2.7.1, using rabbit anti-ZO-1 polyclonal antibody as the primary antibody.

Oxidative Stress: Intracellular reactive oxygen species (ROS) levels were detected using the DCFH-DA fluorescent probe method. After exposure, cells were incubated with 10 μmol/L DCFH-DA probe (Dojindo, Japan) for 30 min at 37 °C in the dark, washed twice with HBSS, and immediately observed under a fluorescence microscope for image acquisition.

Cell Viability and Inflammatory Cytokines: Cell viability and secretion levels of IL-6 and IL-8 were assessed using the methods described in Section 2.6.

### 2.8 Data analysis

All experiments were performed with at least three independent biological replicates, and data are presented as mean ± standard deviation. Normality was tested using the Shapiro-Wilk test. For data following a normal distribution, comparisons between two groups were conducted using a two-tailed Student’s t-test; for data not following a normal distribution, comparisons between two groups were performed using the Mann-Whitney U test. A *p*-value <0.05 was considered statistically significant. Statistical analyses were performed using SPSS 17.0 software, and graphs were generated using OriginPro 2024 software.

## 3 Results and discussion

### 3.1 Construction and flow distribution analysis of the tracheobronchial tree model

**FIGURE 2:**
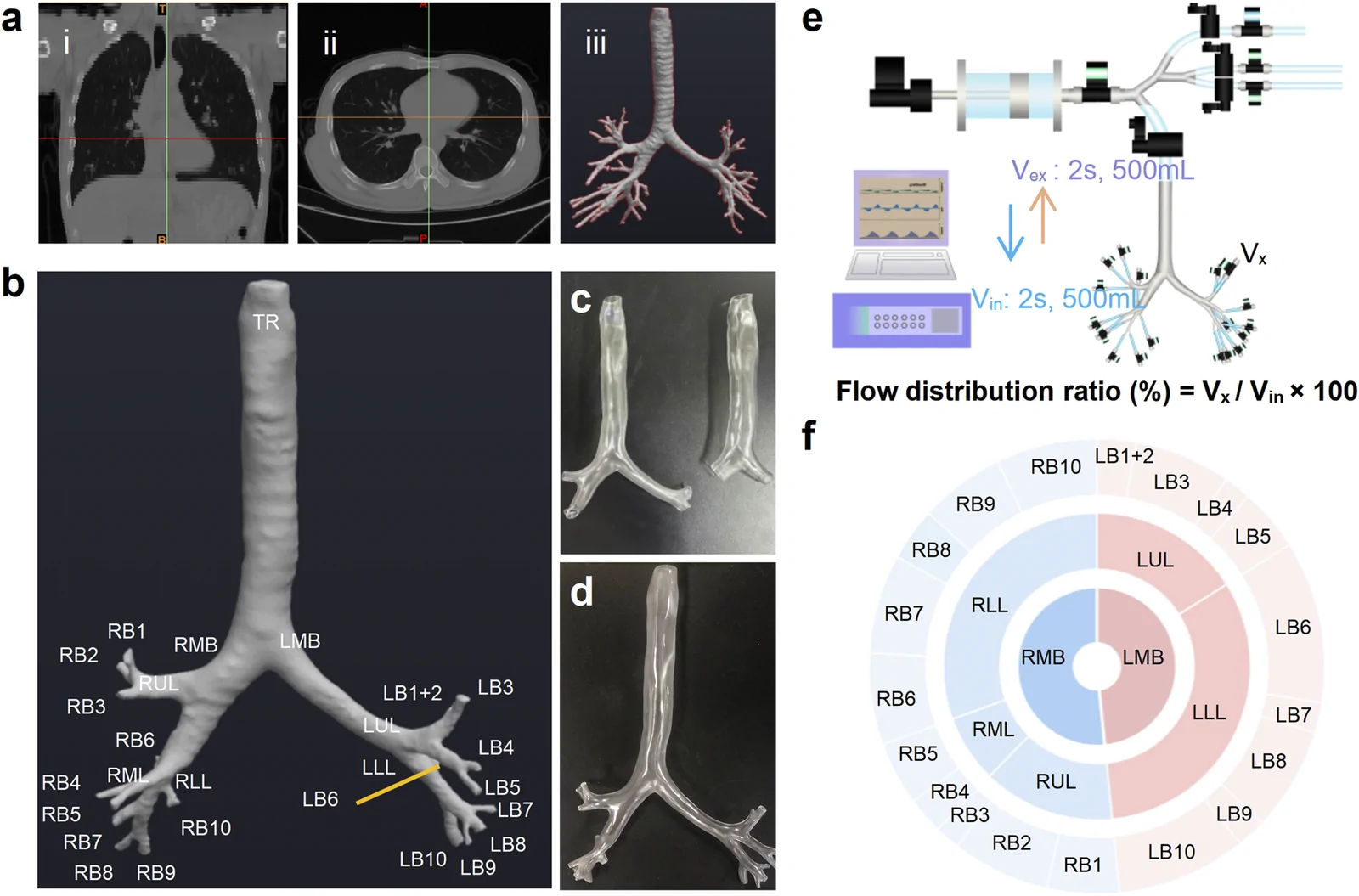
Construction and flow distribution analysis of the tracheobronchial tree model. **(a)** Tracheobronchial tree. (i) coronal chest CT images of healthy adult male, (ii) axial chest CT images of healthy adult male, (iii) three-dimensional reconstructed models of tracheobronchial trees (G0-G6). **(b)** Structural models of tracheobronchial trees including trachea (G0), main bronchi (G1), lobar bronchi (G2), and segmental bronchi (G3) and anatomical nomenclature of hierarchical branches. **(c)** Photographs of physical 3D-printed specimens of tracheobronchial tree models spanning G0-G2 and G0-G1 respectively. **(d)** Photographs of physical 3D-printed specimens of tracheobronchial tree models spanning G0-G3. **(e)** Experimental schematic for airflow distribution ratio measurement. **(f)** Airflow distribution across bronchial generations.

To overcome the limitations of traditional simplified airway models, an anatomically accurate tracheobronchial tree model was developed through 3D reconstruction of chest CT scans (coronal and axial views) from healthy adult males using Mimics software (Figure 2a). Following established morphological standards (Huang et al., 2019; Javed et al., 2023a; Javed et al., 2023b; Wang et al., 2018) and accounting for 3D printing precision requirements, the final model (Figure 2b) encompassed the trachea (G0), main bronchi (G1), lobar bronchi (G2), and segmental bronchi (G3). The 3D-printed model exhibits characteristic asymmetric branching patterns: a 1 (main)-3 (lobar)-10 (segmental) configuration in the right bronchus versus a 1-2-9 pattern in the left bronchus. The 3D-printed physical model demonstrated exceptional fidelity in replicating the morphological characteristics of the original 3D reconstruction (Figures 2c,d). Quantitative measurements of internal branch diameters (Table 2) showed remarkable consistency with reference anatomical data reported by (Mi et al., 2015; Liu Y et al., 2024), confirming the model’s geometric accuracy in both structural configuration and dimensional parameters. This precisely engineered tracheobronchial tree model, with its hierarchical branching architecture, serves as an optimal experimental platform for investigating generation-specific airflow dynamics and aerosol transport phenomena.

**TABLE 2:** Inner diameter measurements (millimeters) for each generation of the physical 3D-printed tracheobronchial tree specimen, including the trachea (G0), main bronchi (G1), lobar bronchi (G2), and segmental bronchi (G3).

Name	ID	Name	ID	Name	ID	Name	ID (millimeters)
TR	11.13–15.45	RMB	10.97–15.1	RUL	5.06–9.93	RB1	2.03
						RB2	1.89
						RB3	0.81
				RML	2.91–3.86	RB4	0.6
						RB5	0.3
				RLL	5.06–9.93	RB6	2.34
						RB7	1.6–2.1
						RB8	0.5
						RB9	0.69–1.22
						RB10	1.8–2.46
		LMB	7.02–8.47	LUL	4.3–11.5	LB1+2	0.7
						LB3	1.45–2.16
						LB4	0.9
						LB5	0.6
				LLL	8.77–9.1	LB6	2.9–3.76
						LB7	0.2
						LB8	0.9–1.3
						LB9	0.2
						LB10	2.47–2.95

Based on this precisely engineered airway model with hierarchical branching structure, the airflow distribution characteristics within the asymmetric bronchial tree under physiological tidal volume conditions (500 mL) were quantitatively characterized. The results revealed a significant “right-sided predominance” in airflow distribution, with the flow rate in the right main bronchus (51.6% ± 0.1%) being significantly higher than that in the left (48.4% ± 0.1%) (*p <* 0.05), while maintaining good flow conservation across all bronchial generations (Figures 2e,f). These experimental findings are highly consistent with computational fluid dynamics (CFD) simulation results reported by (Qi et al., 2017). Notably, this airflow distribution phenomenon resulting from anatomical asymmetry cannot be replicated in traditional straight-through or cloud-settling exposure systems, as these systems employ empty chamber designs lacking airway structures to guide airflow distribution (Bannuscher et al., 2022; Lenz et al., 2009).

The right-dominant airflow pattern reveals an often-overlooked issue in inhalation toxicology: the actual exposure dose differs across airway regions. Morphometric measurements have confirmed that the right main bronchus is wider, shorter, and more vertical than the left (Kim et al., 2014), causing the right lung to receive a higher proportion of particles under the same tidal volume. More importantly, aerosols are complex mixtures; different chemical constituents, due to their distinct particle sizes and diffusion behaviors, undergo region-selective deposition under heterogeneous airflow, leading to spatially targeted toxicity. Conventional cloud-settling systems lack airway branching structures, so particles settle uniformly by gravity alone; even increasing the total dose several-fold cannot reproduce the dose disparity between the right and left lungs or among different bronchial regions, nor can it simulate the targeted distribution of multicomponent mixtures. Epidemiological data provide clinical support for this mechanism. A Japanese registry study of 36,502 lung cancer patients reported right-sided tumors in 60% and left-sided in 40% (Michihata et al., 2025). In their discussion, the study proposed that the higher incidence of right-sided lung cancer might be related to anatomical features of the right lung, such as its larger volume due to three lobes versus two on the left, and the wider, shorter, and smaller branching angle of the right main bronchus. Based on these findings, the central question in inhalation toxicology is not how much dose is inhaled, but rather which region receives which constituents at what dose. The system, through its anatomically accurate airway model and multi-interface design, achieves experimental resolution of regional dose disparities and selective deposition of aerosol components *in vitro*. This necessity underscores the value of such complexity-rather than merely adjusting the total dose in a cloud-settling system.

Building upon this foundation, the system further incorporates a multi-interface design supporting connection of cell exposure units to the oral cavity, nasal cavity, and different levels of bronchial branches (G0-G3). This design enables investigation of deposition dose differences of aerosols through different inhalation routes (oral/nasal/oral + nasal breathing) and across various airway regions (different bronchial generations) within the same respiratory cycle, thereby providing a critical structural foundation for subsequent studies on airway region-specific exposure. The subsequent cell exposure experiments (see Section 2.6, Section 2.7) were precisely based on this design, achieving accurate exposure assessment of specific airway regions. The precise flow distribution data obtained in this study not only provide critical parametric benchmarks for respiratory airflow dynamics modeling but also establish a theoretical foundation for regional dose normalization in *in vitro* exposure experiments.

Although this study has quantitatively revealed airflow distribution characteristics within the asymmetric bronchial tree through experimental means and provided key parametric benchmarks for respiratory dynamics modeling, comprehensive understanding of aerosol behavior in complex airways still requires CFD dynamics simulations. CFD can resolve transport trajectories of particles with different sizes, predict wall deposition hotspots induced by inertia and turbulence, and quantify dose uniformity across cell exposure surfaces—parameters essential for extrapolating *in vitro* exposure results to *in vivo* risk assessment. In subsequent studies, high-fidelity computational fluid dynamics meshes will be constructed based on the precisely reconstructed 3D-printed airway model, incorporating experimentally measured inlet flow velocity waveforms (as shown in Figure 2), to systematically investigate deposition patterns of cigarette smoke and environmental particles within generations 0-3 of the bronchi. Furthermore, a closed-loop interface between computational fluid dynamics simulations and the physical system is planned, utilizing real-time aerosol tracking algorithms to achieve sub-millimeter precision particle deposition prediction, and ultimately validate simulation results using experimental deposition data (as shown in Figure 2). This complementary “experimental-simulation” research strategy will provide a powerful tool for in-depth understanding of region-specific aerosol deposition mechanisms and optimization of exposure dosimetry.

### 3.2 System performance validation: dynamic breathing simulation and recreation of smoking patterns

Building upon the validated static anatomical model, experimental validation of the system’s dynamic breathing simulation capability was further conducted. The system supports free programming of inhalation, breath-hold, and exhalation modes, with independently adjustable parameters including breathing waveform (5 selectable types) (Appleton et al., 2015), tidal volume (0–1000 mL), respiratory rate (0–20 breaths/min), inhalation/exhalation duration (0–60 s), breath-hold interval (0–10 min), CO_2_ concentration (0–100%), and airflow direction. Based on this, the evaluation focused on its accuracy in replicating classic small-loop smoking with breathing pattern, classic large-loop smoking with breathing pattern, and personalized smoking with breathing pattern.

**FIGURE 3:**
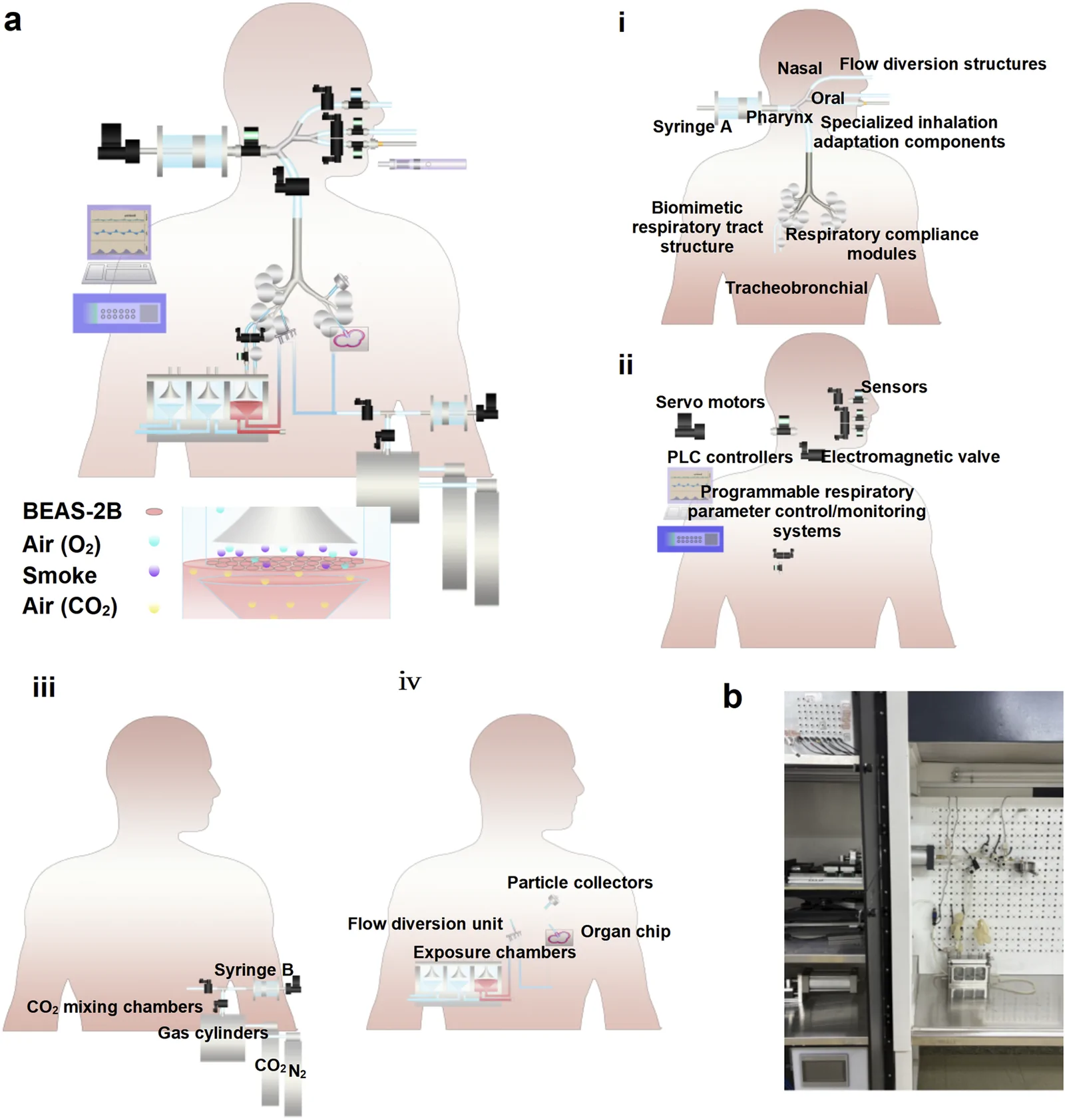
Bionic breathing-type airway exposure system. **(a)** Components of the bionic breathing-type airway exposure system: (i) airway structural unit:biomimetic respiratory tract structure (oral/nasal cavities, pharynx, tracheobronchial tree, and respiratory compliance modules), specialized inhalation adaptation components, flow diversion structures, and syringe A; (ii) dynamic control and monitoring unit: PLC controllers, servo motors (for syringe actuation), electromagnetic valve arrays, programmable respiratory parameter control/monitoring system, and sensors; (iii) exhaled gas environment simulation unit: gas cylinders, CO_2_ mixing chambers, syringes B; (iv) multi-scenario exposure interface: serial tubing networks, exposure chambers (commercially available or custom-designed exposure chambers, organ chip), and particle collectors. **(b)** Physical bionic breathing-type airway exposure system.

**FIGURE 4:**
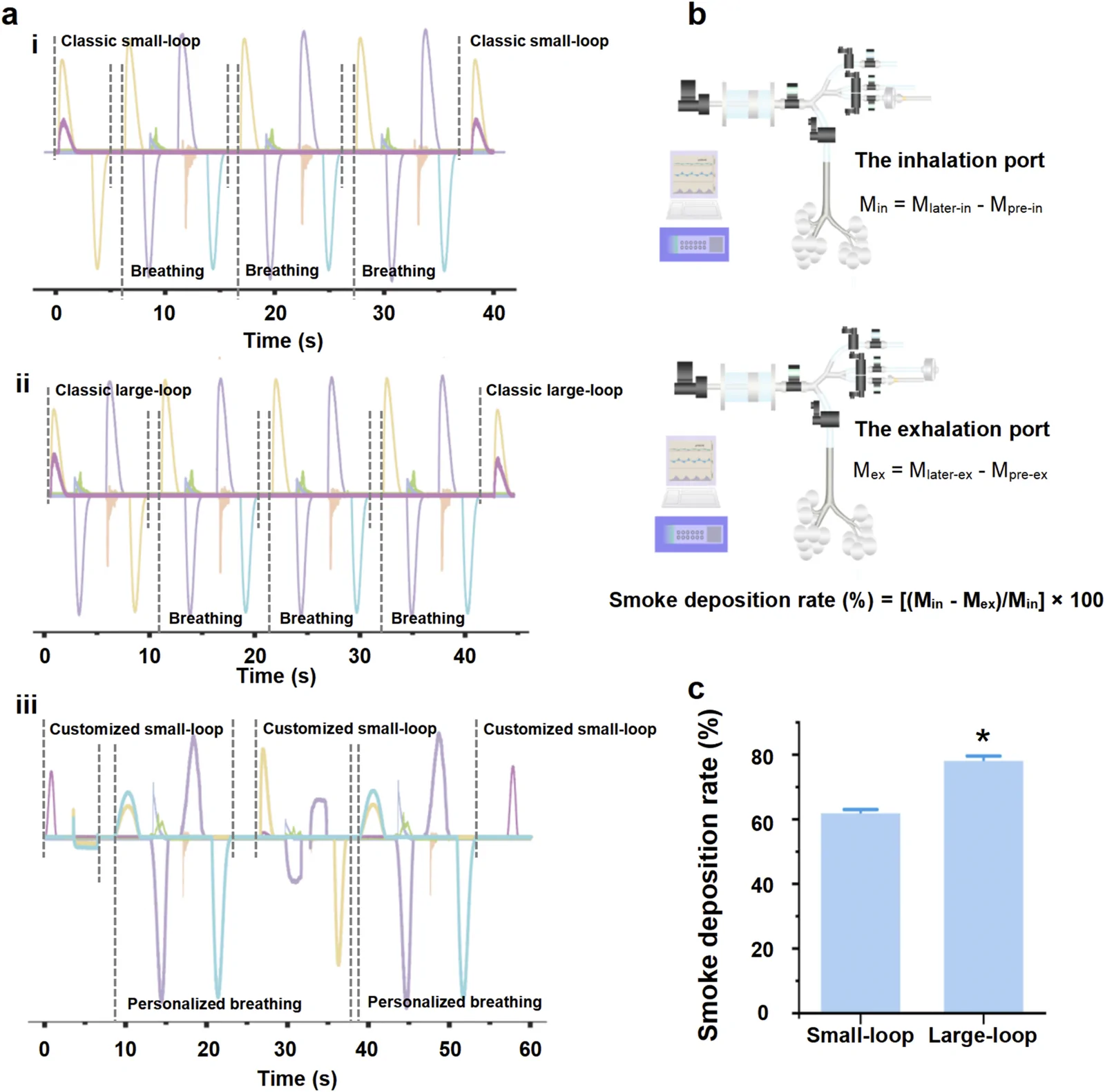
Representative time-flow waveforms under three characteristic inhalation-smoking patterns and physical characterization of the biomimetic respiratory tract structure. **(a)** Schematic representation of time-flow waveforms illustrating the timing and morphological characteristics of three inhalation-smoking patterns: (i) classic small-loop smoking with breathing pattern; (ii) classic large-loop smoking with breathing pattern; (iii) personalized smoking with breathing pattern. Waveforms are shown as relative values to emphasize temporal sequencing and flow profile morphology (e.g., sine wave, square wave, triangular wave) rather than absolute flow magnitudes. **(b)** Experimental schematic for airflow distribution ratio measurement. **(c)** Smoke deposition rate under the classic small-loop vs. large-loop smoking with breathing (n = 3, mean ± SD, *p < 0.05).

Validation results (Figure 3; Table 3; Figure 4) demonstrate that the system exhibits excellent physiological biomimetic characteristics across four key dimensions: respiratory timing, flow control, physiological parameter simulation, and microenvironment maintenance.

**TABLE 3:** Experimentally measured system performance metrics (flow rate, time, CO_2_ concentration) under three characteristic inhalation-smoking patterns.

Parameters	Set value	Measured value	Relative deviation(%)
Duration of inhalation/exhalation (s)	1.5	1.504 ± 0.004	−0.27
	2	2.008 ± 0.002	−0.40
	3	3.008 ± 0.002	−0.27
Pause/breath-holding time (s)	0.2	0.219 ± 0.008	−8.68
	1	1.01 ± 0.002	−0.99
	2	2.01 ± 0.006	−0.50
	3	2.99 ± 0.006	0.33
Tidal volume(mL)	300	300.35 ± 0.19	−0.12
	500	500.53 ± 0.24	−0.11
	510	510.50 ± 0.24	−0.10
	800	800.80 ± 0.43	−0.10
	830	830.83 ± 0.83	−0.10
Mouth inhalation flow (mL)	35	35.31 ± 0.20	−0.88
	55	55.17 ± 0.41	−0.31
Nasal inhalation flow (mL)	465	465.32 ± 0.18	−0.07
	445	445.01 ± 0.16	0.00
The amount of CO_2_ entering the exposed chamber (mL)	10	9.92 ± 0.34	0.81
	30	30.21 ± 0.42	−0.70
CO_2_ generation concentration (%)	16	16 ± 0.16	0
	30	30 ± 0.4	0

Timing control precision (Table 3): The system achieved millisecond-level coordinated control of the “inhalation–breath hold–exhalation” cycle with smoke exposure. Measured data show that for programmed inhalation/exhalation durations across different modes (1.5 s, 2 s, 3 s), timing errors were ≤0.40%; for programmed breath-hold/pause durations (0.2 s, 1 s, 2 s, 3 s), control errors were ≤ ± 0.99% for all breath-hold periods, except for the 0.2 s brief pause which exhibited a relative deviation of −8.68% due to mechanical response limits. This precision ensures that within each inhalation phase, the system can synchronously execute multi-channel gas delivery according to the programmed sequence, such as simultaneously completing oral smoke inhalation (35 mL) and nasal air inhalation (465 mL) within a 2 s inhalation window (Figure 4a, Supplementary Materials 8a,b), precisely synthesizing a 500 mL tidal volume. The synchronization error between the breathing cycle and the smoke generation unit was ≤10 m, fundamentally eliminating dose deviations caused by timing mismatch in traditional models and ensuring that the timing characteristics of *in vitro* exposure closely match authentic human respiratory cycles.

Flow control precision (Table 3): Building on precise timing control, the system demonstrated exceptional flow control capability. For programmed tidal volumes (300 mL, 500 mL, 510 mL, 800 mL, 830 mL), measured flow deviations were ≤ ±0.12%; for oral smoke intake (35 mL, 55 mL) and nasal air intake (445 mL, 465 mL), control deviations were ≤ ±0.88% and ≤ ± 0.07%, respectively. This confirms the system’s ability to precisely achieve simultaneous multi-channel gas diversion and synthesis within the same inhalation window, providing a reliable dosimetry foundation for regional toxicity studies that distinguish between oral and nasal exposure.

Physiological parameter simulation capability: Based on precise time-flow control, the system further achieved extensive physiological parameter simulation. Regarding waveform generation, it successfully reproduced five physiologically relevant breathing waveforms (Figure 4a and Supplementary Material 8), including respiratory wave, sine wave, bell-shaped wave, triangular wave, and square wave, which can be freely selected across different modes according to experimental requirements. For example, the respiratory wave simulates natural breathing, the square wave simulates volume-controlled mechanical ventilation, and the triangular wave simulates COPD patient ventilation (flow rate rises to peak and then declines). In terms of respiratory parameter adjustment (Table 3), tidal volume can be stably regulated within the 300–830 mL range (system range 0–1000 mL), and respiratory rate supports full-range setting up to ≤20 breaths/min. Regarding dynamic regulation of gas composition, the system can precisely inject CO_2_ at specific phases of the breathing cycle. In tidal breathing mode, 10 mL of 16% CO_2_ is injected before exhalation, achieving a final concentration of 3.57% in the exposure chamber to simulate normal physiological exhaled levels; in personalized smoking mode, 30 mL of 30% CO_2_ is injected during the gas exchange phase to simulate high CO_2_ exhalation characteristics under pathological conditions. The measured deviation for CO_2_ injection volume was ≤ ±0.81%, and the generated concentrations were fully consistent with set values (16% ± 0.16%, 30% ± 0.4%). These results demonstrate that the system supports independent programming of all parameters including breathing waveform, tidal volume, and CO_2_ injection timing and concentration, enabling flexible simulation of multi-scenario respiratory characteristics ranging from normal physiology to pathological states.

Microenvironment maintenance: During dynamic breathing processes, the maintenance of cell viability depends on the stability of the gas environment within the exposure chamber. Through real-time monitoring and feedback control, the system ensures that O_2_ concentration is consistently maintained within the physiological range of 16–21%, providing sufficient oxygen supply for cellular metabolism. Simultaneously, despite pulsed CO_2_ injection and removal during the breathing cycle, the system maintains steady-state CO_2_ concentration in the exposure chamber at levels suitable for cell growth, such as 3.57% ± 0.1% in tidal breathing mode. The CO_2_ regulation range covers 0–100%, accommodating diverse requirements from normal culture to high CO_2_ pathological model construction. This stable microenvironment control system provides reliable assurance for cell survival and functional maintenance during long-term dynamic exposure experiments.

Pattern recreation verification (Figure 4a, Supplementary Material 8): To systematically evaluate the system’s programmability and physiological biomimetic performance, three characteristic smoking with breathing patterns were designed and validated, each operating in a cyclic sequence manner, fully demonstrating the system’s ability to control complex breathing programs during long-term experiments.

Classic small-loop smoking with breathing pattern operates with “1 small-loop smoking +3 tidal breaths” as one complete cycle, running cyclically. At cycle initiation, the system executes the small-loop smoking procedure: within a 2 s inhalation window, simultaneous oral inhalation of 35 mL smoke and nasal inhalation of 465 mL air synthesizes a 500 mL tidal volume; after a 1 s breath hold, 500 mL is exhaled nasally. Subsequently, three tidal breaths are executed consecutively: nasal inhalation of 500 mL air → 0.2 s pause → gas enters alveolar module and exposure chamber → injection of 10 mL 16% CO_2_ → oral exhalation of 510 mL. Upon completion, the system automatically proceeds to the next cycle, repeating the small-loop smoking followed by three breaths.

Classic large-loop smoking with breathing pattern employs the same cyclic structure, operating with “1 large-loop smoking +3 tidal breaths” as one cycle. Large-loop smoking integrates a gas exchange phase based on small-loop smoking: after oronasal smoke inhalation (35 mL oral smoke +465 mL nasal air), gas enters the alveolar module and exposure chamber, with simultaneous CO_2_ injection simulating tissue exhalation, and finally nasal exhalation, enabling smoke to undergo the complete physiological process of “inhalation–exchange–exhalation.” The three tidal breaths within the cycle are consistent with the classic small-loop pattern.

Personalized smoking with breathing pattern demonstrates the system’s higher-order programming flexibility, enabling independent programming of waveforms, durations, and breath-hold times for different phases throughout the entire operational sequence. The typical sequence executed in this validation was: first, personalized small-loop smoking—300 mL smoke inhalation (sine wave) within a 1.5 s inhalation window, 2 s breath hold, 300 mL exhalation (square wave) within a 3 s exhalation window; then, personalized breathing—800 mL mixed gas inhalation (bell-shaped wave) within a 3 s inhalation window, gas exchange phase with 30% CO_2_ injection (sine wave) and 830 mL withdrawal (triangular wave), 830 mL exhalation (triangular wave) within a 3 s exhalation window; followed by personalized large-loop smoking—oral inhalation of 55 mL smoke and nasal inhalation of 445 mL air within a 2 s inhalation window, gas exchange phase using square wave, and finally 510 mL nasal exhalation; concluding with another personalized breathing sequence. Throughout the entire operational process, breathing waveforms, inhalation/exhalation durations, and breath-hold times for each phase can be independently programmed, achieving complex and varied combinations of smoking and breathing sequences.

The successful recreation of the above three pattern types demonstrates that the system not only supports precise control of single-cycle respiratory events but can also stably execute complex cyclic sequences during long-term operation, allowing independent programming of respiratory parameters for each phase within the sequence. From classic smoking behavior to personalized pathological state simulation, the system can accurately reproduce corresponding respiratory characteristics.

Compared to existing systems, the platform exhibits pronounced differentiated advantages. Existing cloud-settling exposure systems (such as the VITROCELL® Cloud series) rely on passive gravitational settling, with aerosols introduced directly into an empty chamber, lacking simulation of respiratory cycles (Bannuscher et al., 2022; Lenz et al., 2009). The present system, in contrast, integrates anatomical airway structures and dynamic breathing cycles to simulate the human “active inhalation” process. Compared with Chen et al.'s system (Chen et al., 2024), the present system demonstrates marked advantages in parametric combination diversity and adaptability to specific populations, achieving millisecond-level synchronization between smoke inhalation and respiratory cycles. This successfully addresses key limitations of conventional models, including asynchrony between respiration and exposure, oversimplified airway geometry, and lack of coordinated oral-nasal exposure pathways. Furthermore, configurable waveform and exhaled CO_2_ concentration settings enable the system to simulate respiratory-smoking characteristics under diverse pathophysiological states (e.g., COPD (Davies et al., 2024; Wang et al., 2021a), mechanical ventilation (Mora Carpio and Mora, 2026), hypercapnia (Schellongowski et al., 2015; Almanza-Hurtado et al., 2022)).

In addition to the systems discussed above, several other biomimetic exposure platforms have been reported that share conceptual similarities with the present approach. (Lizal et al. (2012) developed a realistic digital upper airway model based on CT data and fabricated multiple physical models for flow and deposition measurements, providing valuable geometries for CFD simulations and experimental validation. However, their model did not include an oral cavity and was not integrated with dynamic breathing control or cell culture exposure interfaces. Steiner et al. (2020) introduced the InHALES (Independent Holistic Air-Liquid Interface Exposure System), which mechanically simulates the entire human respiratory tract and can actively breathe, operate inhalers, or puff on tobacco products while exposing cell cultures at the air-liquid interface. Although conceptually innovative, the InHALES employs idealized airway geometries (based on Weibel models) rather than patient-specific CT-reconstructed anatomy, and it does not support independent programming of oral versus nasal inhalation routes or branch-level deposition resolution. Sarles and colleagues developed a Biomimetic Aerosol Exposure System (BAES) and systematically characterized its wall shear stress (Emma Sarles et al., 2024) and mass distribution (Sarles et al., 2024), demonstrating the influence of puff topography and post-puff inhalation on aerosol deposition. While their work provides important insights into dosimetry, the BAES lacks anatomically accurate branching geometry and has not been integrated with complex cell models for biological endpoint assessment. Benam and collaborators created a biomimetic smoking robot compatible with microfluidic organ chips (Benam et al., 2020) and later extended it for real-time submicron and microparticle analysis (Kaiser et al., 2021), enabling dynamic smoke exposure of living airway epithelium under physiological breathing patterns. Their work represents a major advance in integrating smoking topography with organ-on-a-chip technology. Nevertheless, the airway geometry in their system is simplified to microchannels rather than a full-scale anatomically accurate bronchial tree, and independent control of oral versus nasal inhalation is not implemented. In contrast, the present platform integrates these features into a single system: (1) a CT-derived G0-G3 anatomically accurate airway model with validated geometric fidelity (Table 2), (2) independent programming of oral and nasal inhalation routes, (3) a multi-interface design enabling branch-specific exposure resolution (e.g., connecting cell culture units to individual bronchial branches such as RB1 vs. LB2), and (4) a physiological CO_2_ exchange module that dynamically maintains cellular microenvironment stability during cyclic breathing. The integration of these features enables the system to address several limitations of existing biomimetic exposure platforms and offers an alternative tool for region-specific inhalation toxicology and drug screening.

Leveraging these technological advantages, the classic “small-loop” (oral deposition) and “large-loop” (deep lung inhalation) patterns of smoking behavior were precisely replicated (Figures 4b,c). Quantitative analysis revealed that the mean deposition rate in large-loop mode reached 78.0% ± 1.6%, significantly exceeding the 61.7% ± 1.3% observed in small-loop mode (*p* < 0.05). This difference aligns with trends reported in human studies (Wang et al., 2021b; Asgharian et al., 2024; Baker and Dixon, 2006), confirming that the anatomically accurate airway model integrated into the system can effectively capture regional deposition variations resulting from different inhalation behaviors.

The deposition rates observed in this study fall at the lower end of the population-reported range, which may be attributed to (Oberdörster and Pott, 1987): (1) the structural complexity of the three-dimensional airway system; (2) variability in cigarette combustion parameters; (3) individual differences in inhalation depth; and (4) the material properties of the airway model surface, such as wettability and roughness, which can influence particle adhesion during aerosol transport. Although the 3D-printed model was fabricated from transparent photopolymer resin and polished to a smooth finish, its surface properties—such as wettability and roughness—may differ from those of native mucus-coated epithelium, potentially influencing particle deposition. Nevertheless, these results compellingly demonstrate that the system not only simulates the breathing action itself, but also effectively discriminates exposure variations induced by different inhalation behaviors and accurately replicates the characteristic deposition patterns of smoke particles in the respiratory tract. This resolution of inhalation behavior specificity is unattainable in existing traditional exposure systems, as they cannot distinguish whether aerosols are “inhaled and then deposited” or merely “settled” onto the cell surface (Rothen-Rutishauser et al., 2023). It is worth emphasizing that, based on the multi-interface design described in Section 2.1, the system possesses the unique capability to resolve aerosol deposition differences at the branch level. This design enables future studies to quantitatively assess the heterogeneous distribution characteristics of particles across G0-G3 bronchi—for example, the regional deposition heterogeneity driven by anatomical asymmetry (such as the “right-sided predominance” of airflow shown in Figures 2e,f) can potentially be directly validated experimentally in this system. This ability to capture deposition characteristics at the airway branch level represents a critical functional breakthrough unattainable in traditional “single-interface exposure” systems. The above system characteristics indicate that the platform not only simulates overall inhalation behavior but also provides potential experimental approaches for subsequent investigations of dose-response relationships in different airway regions. Therefore, this system offers an unprecedented tool for studying personalized inhalation exposure in specific populations (e.g., COPD patients) and regional particle deposition processes in the human airways.

### 3.3 Platform feasibility validation using the BEAS-2B cell model

**FIGURE 5:**
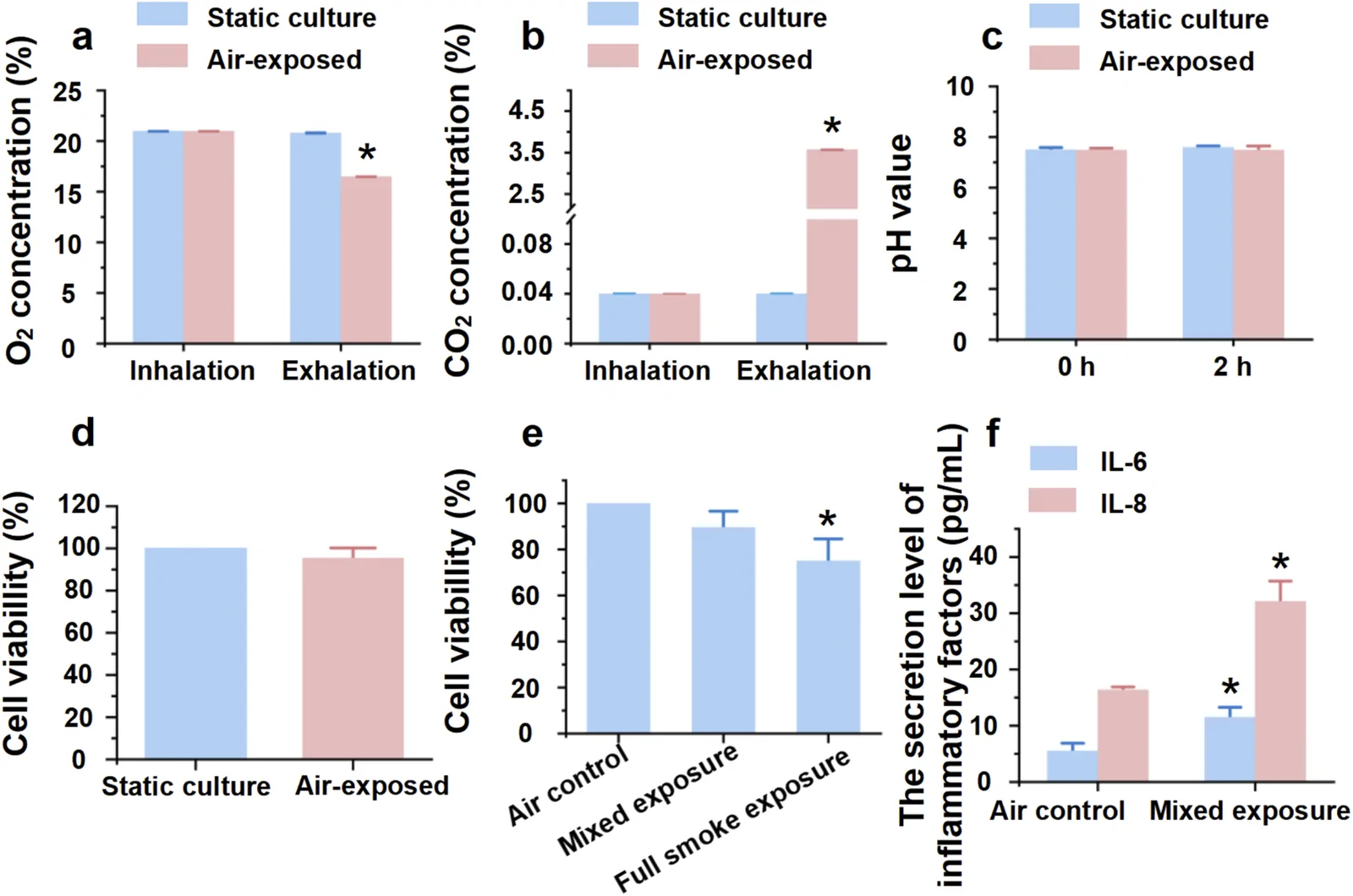
Simulated environmental and cellular responses under breathing and smoking scenarios. **(a)** Variations in O_2_ concentration within the culture chamber during simulated static culture and air-exposed conditions (n = 3, mean ± SD, *p < 0.05). **(b)** Variations in CO_2_ concentration within the culture chamber during simulated static culture and air-exposed conditions (n = 3, mean ± SD, *p < 0.05). **(c)** Changes in culture medium pH during simulated static culture and air-exposed conditions. **(d)** Viability of BEAS-2B cells in the culture chamber under simulated static culture and air-exposed conditions. **(e)** Viability of BEAS-2B cells in the culture chamber under classic large-loop smoking cycle scenarios (n = 3, mean ± SD, *p < 0.05). **(f)** Expression levels of IL-6 and IL-8 in BEAS-2B cells under classic large-loop smoking cycle scenarios (n = 3, mean ± SD, *p < 0.05).

Following integration of the system with the BEAS-2B cell model, its biological effects were further evaluated. First, the system successfully simulated a physiological air-blood exchange microenvironment (Figure 5). During the inhalation phase, cells were exposed in the gaseous environment to air containing 21% O_2_/0.04% CO_2_; during the exhalation phase, they were exposed to air containing 3.57% ± 0.01% CO_2_/16.49% ± 0.05% O_2_. Notably, despite these gaseous composition changes, the liquid environment surrounding the cells maintained stable pH (7.49 ± 0.16) and cell viability, consistent with the characteristics of the human airway microenvironment. Building on this, dose-response experiments (Figures 5e,f) further revealed that increasing puff counts (mixed exposure vs. full smoke exposure, equivalent to exposure doses of 3.45 puffs vs. 6.90 puffs) significantly reduced BEAS-2B cell viability by 25.0% (*p* < 0.05), while simultaneously increasing IL-8 secretion (from 16.37 ± 0.53 to 32.10 ± 3.62 pg/mL) and IL-6 secretion (from 5.51 ± 1.39 to 11.51 ± 1.76 pg/mL), respectively (*p* < 0.05) (Hua et al., 2020; Azzopardi et al., 2015; Li F et al., 2023). The system successfully detected dose-dependent cytotoxic effects and inflammatory responses (e.g., significant elevation of IL-8) induced by cigarette smoke aerosol exposure, which closely mirror the pathological features of smoking-associated respiratory diseases (Ball et al., 2023; Powers and Dhamoon, 2026; Li P et al., 2023).

Notably, compared to traditional static exposure models (which induced effects at exposure doses of 0.96 and 4.56 puffs), the system required higher smoke exposure doses (3.45 and 6.90 puffs) to elicit equivalent levels of cytotoxicity. This difference is by no means a design flaw, but rather a direct reflection of the fundamental paradigm shift between the present system and existing systems. Existing systems (such as VITROCELL® Cloud) rely on passive gravitational settling, with aerosols introduced directly into an empty chamber; theoretically, 100% of particles eventually settle onto the cell surface, which may lead to overestimation of the actual dose received by the cells (Bannuscher et al., 2022; Lenz et al., 2009), thereby overestimating the immediate toxicity of smoke. In contrast, the present system integrates anatomical airway structures and dynamic breathing cycles to simulate the human “active inhalation” process: a portion of the particles deposit in the upper airways before reaching the cells, while another portion is expelled from the system during the exhalation phase. Therefore, achieving the same cellular effects as static systems requires higher nominal exposure doses—a finding that precisely reflects the physiological reality of *in vivo* exposure. This finding underscores the importance of incorporating respiratory dynamics into inhalation toxicology, demonstrates that the system overcomes the oversimplification limitations of static exposure systems (Ball et al., 2023; Powers and Dhamoon, 2026; Li P et al., 2023; Xi et al., 2020), and effectively discriminates exposure differences arising from breathing patterns themselves, providing a more reliable system for accurately assessing the health risks of environmental pollutants and tobacco products.

It should be noted that although the BEAS-2B cells used in this study were cultured at the air-liquid interface during exposure, they lacked key defense mechanisms such as cilia and mucus layers. Therefore, this model was primarily employed for proof-of-concept validation of the system’s basic functionality and the initial biological responses to smoke exposure (e.g., cytotoxicity and inflammatory cytokine release).

### 3.4 Smoke exposure assessment using a human induced pluripotent stem cell-derived lung model

**FIGURE 6:**
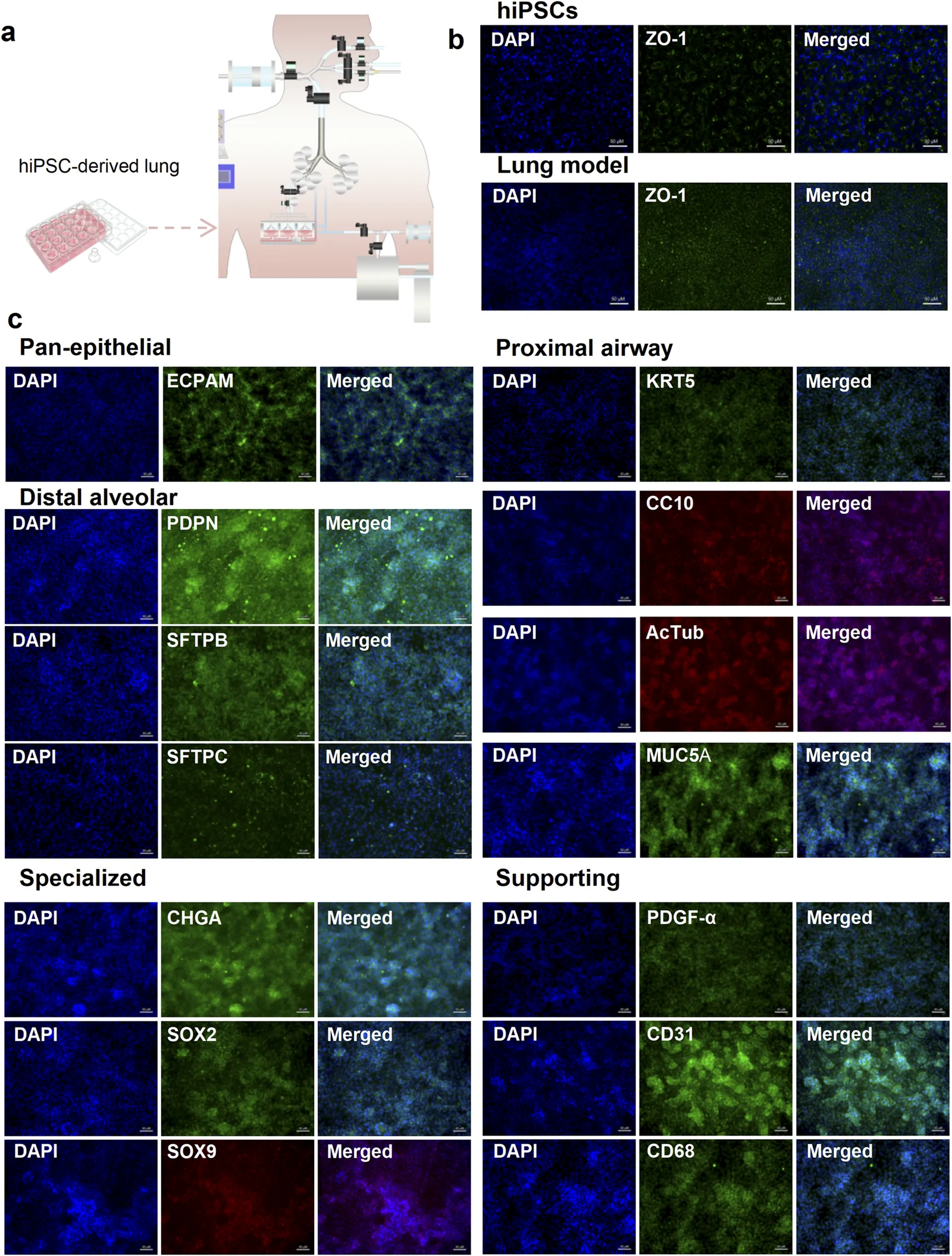
Characterization of the hiPSC-derived lung model. **(a)** Schematic diagram of smoke exposure experiments using the hiPSC-derived lung model. **(b)** Immunofluorescence staining of tight junction protein ZO-1 (green) in the hiPSC-derived lung model; nuclei were counterstained with DAPI (blue). Scale bar = 50 μm. **(c)** Immunofluorescence staining of multiple cell type-specific markers in the hiPSC-derived lung model, including basal cells (KRT5^+^), ciliated cells (ACTUB^+^), Club cells (CC10^+^), goblet cells (MUC5AC^+^), alveolar type I cells (PDPN^+^), alveolar type II cells (SPC^+^/SPB^+^), neuroendocrine cells (CHGA^+^), progenitor cells (SOX2^+^/SOX9^+^), mesenchymal cells (PDGFR-α^+^), endothelial cells (CD31^+^), and macrophage-like cells (CD68^+^). Scale bar = 50 μm.

To overcome the limitations of the BEAS-2B model and gain more physiologically relevant insights into toxicity mechanisms, a human induced pluripotent stem cell (hiPSC)-derived lung model was further introduced. This model can form an airway epithelium-like structure containing multiple lung cell types and establish intercellular protective barriers (Figure 6), thereby providing a more effective platform for studying the effects of smoke exposure on a lung microenvironment that closely mimics real human conditions. As shown in Figure 6, ZO-1 immunostaining reveals continuous tight junction rings, confirming an intact epithelial barrier (Figure 6b). Meanwhile, immunofluorescence staining (Figure 6c) shows positive expression of multiple cell type-specific markers, including KRT5 (basal cells), AcTUB (ciliated cells), CC10 (Club cells), MUC5AC (goblet cells), PDPN (type I alveolar cells), SFTPB/SFTPC (type II alveolar cells), CHGA (neuroendocrine cells), SOX2/SOX9 (progenitor cells), PDGFR-α (mesenchymal cells), CD31 (endothelial cells), and CD68 (macrophage-like cells), confirming the presence of a multicellular, airway-like tissue structure. These features recapitulate the heterogeneous multicellular composition and cell-cell interactions of the human airway, providing a more physiologically relevant environment for smoke exposure studies. Compared to BEAS-2B monolayers, this hiPSC model represents a qualitative leap in cellular complexity, more closely aligning with the “highly physiologically relevant lung model” concept advocated by Rothen-Rutishauser et al. (Rothen-Rutishauser et al., 2023).

**FIGURE 7:**
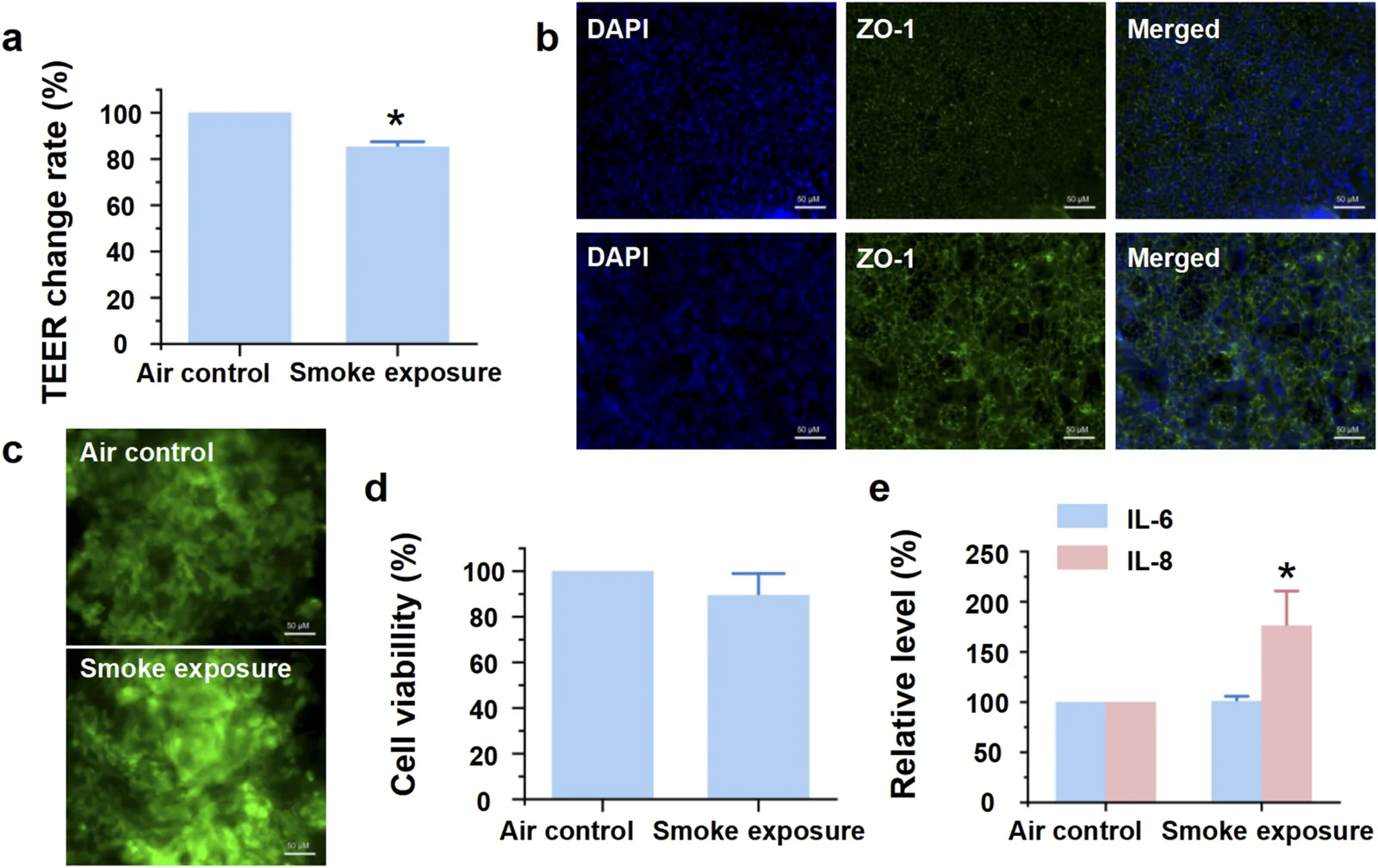
Smoke exposure-induced barrier damage, oxidative stress, and inflammatory responses. **(a)** Changes in transepithelial electrical resistance (TEER) of the hiPSC-derived lung model under classic large-loop smoking cycle scenarios (n = 3, mean ± SD, *p < 0.05). **(b)** Immunofluorescence staining of tight junction protein ZO-1 (green) in the hiPSC-derived lung model following smoke exposure; nuclei were counterstained with DAPI (blue). Scale bar = 50 μm. **(c)** Fluorescence detection of reactive oxygen species (ROS) levels (green) in the hiPSC-derived lung model following smoke exposure. Scale bar = 50 μm. **(d)** Cell viability of the hiPSC-derived lung model assessed by CCK-8 assay following smoke exposure (n = 3, mean ± SD). **(e)** Secretion levels of IL-6 and IL-8 in the hiPSC-derived lung model measured by ELISA following smoke exposure (n = 3, mean ± SD, *p < 0.05).

Following exposure to the “large-loop” smoking pattern on this platform, the hiPSC lung model exhibited biological responses similar to, yet more complex than, those observed in BEAS-2B cells (Figure 7), revealing unique regulatory mechanisms of smoke toxicity within complex cellular populations.

Epithelial barrier function and oxidative stress: Smoke exposure compromised epithelial barrier function in the hiPSC-derived lung model, as evidenced by a significant reduction in TEER values to 85.27% of baseline (*p* < 0.05) and disrupted, diffuse immunofluorescence signals for the tight junction protein ZO-1 (Figures 7a,b). Concurrently, intracellular ROS levels increased significantly (Figure 7c). These findings align with the established role of oxidative stress as a conserved mechanism of smoke-induced epithelial injury, and demonstrate the utility of this hiPSC model in recapitulating key pathological features.

Maintenance of cell viability: Notably, although the hiPSC model received a higher surface area-normalized dose (3.48 puffs/cm^2^) than the doses that induced toxicity in BEAS-2B cells (0.74 and 1.48 puffs/cm^2^, respectively), cell viability did not decrease significantly (Figure 7d). This seemingly paradoxical result may reflect multiple protective mechanisms inherent in the complex lung model: first, progenitor cell populations present in the model, such as basal cells and Club cells, may possess enhanced anti-apoptotic and repair capacities; second, paracrine signaling among multiple cell types (e.g., neurotrophic factors from mesenchymal or macrophage-like cells) may increase overall tissue tolerance to oxidative stress; and third, the established tight junctions themselves (Figure 7b) may provide a more effective physical barrier than monolayers, reducing direct cellular contact with smoke components.

Specificity of the inflammatory response: Regarding inflammatory cytokine release, the hiPSC lung model exhibited a distinctly different pattern from BEAS-2B cells. While BEAS-2B cells showed concomitant upregulation of IL-6 and IL-8 following smoke exposure (Figure 5f), the hiPSC model displayed a significant increase only in IL-8, with no significant change in IL-6 levels (Figure 7e). This differential inflammatory cytokine release pattern carries important biological implications: as a potent neutrophil chemoattractant, the isolated elevation of IL-8 may reflect an early, innate immune response of the airways to smoke exposure; conversely, the absence of IL-6 activation suggests the presence of negative feedback regulatory mechanisms in the complex model that prevent excessive inflammatory responses. This phenomenon likely stems from complex interactions among the various cell types within the model—for instance, CD68^+^ macrophage-like cells may suppress IL-6 production through the release of anti-inflammatory factors such as IL-10, while preserving IL-8-mediated neutrophil recruitment function (Rothen-Rutishauser et al., 2023). Such fine-tuned regulation is unattainable in simple BEAS-2B monolayers, underscoring the necessity of studying toxicity mechanisms in complex, physiologically relevant models.

In summary, the exposure results from the hiPSC lung model not only validate the application potential of the platform in more complex biological systems but also reveal a key insight into smoke exposure toxicology: the complexity of the cellular population dictates the complexity of the biological response. The “typical” toxicity response observed in BEAS-2B monolayers (cell death + broad-spectrum inflammation) is finely regulated in the more physiologically relevant hiPSC model into a pattern of “barrier damage + oxidative stress + selective inflammation (IL-8).” This pattern more closely mirrors the pathological features of “neutrophilic inflammation accompanied by epithelial barrier dysfunction” observed in the airways of smokers. Therefore, combining the high-fidelity exposure capabilities of the platform with physiologically relevant cell models opens new avenues for elucidating the toxicity mechanisms of real-world complex mixtures such as cigarette smoke and air pollutants.

## 4 Conclusion

In this study, a bionic breathing-type airway exposure system was successfully constructed that integrates an anatomically precise airway model, millisecond-synchronized dynamic breathing control, independently controlled oral and nasal exposure interfaces, and a physiological gas exchange module. This system systematically overcomes the inherent limitations of existing *in vitro* systems regarding anatomical structure, respiratory dynamics, and exposure route singularity. Benefiting from its biomimetic design, the system accurately recapitulates airflow distribution characteristics within the asymmetric bronchial tree and precisely simulates smoking-specific small-loop and large-loop patterns and their decisive influence on particle deposition efficiency, thereby achieving the precise simulation of the complete process of “how aerosols are inhaled, where they deposit, and how much deposits” in an *in vitro* model.

After integration with BEAS-2B cell models, the system successfully detected dose-dependent cytotoxicity and inflammatory responses induced by cigarette smoke exposure, and revealed the significant regulatory role of dynamic breathing processes on nominal exposure doses—a finding that underscores the importance of incorporating respiratory dynamics into inhalation toxicology. Furthermore, by incorporating a hiPSC-derived complex lung model, the system uncovered a finely regulated pattern of “epithelial barrier damage—oxidative stress activation—selective inflammation (IL-8↑)” induced by smoke exposure in a near-physiological microenvironment, a profile that more closely resembles the pathological features of smokers' airways compared to traditional monolayer cell models.

In summary, through its bionic breathing functionality, this system establishes a new paradigm for *in vitro* inhalation exposure research, providing an innovative tool for elucidating the health risks of complex mixtures such as tobacco smoke and air pollutants, and demonstrating broad application prospects in environmental health risk assessment and the development of personalized inhalation therapies.

## Data Availability

The raw data supporting the conclusions of this article will be made available by the authors, without undue reservation.
