# Supplementary material for: Bionic breathing-type airway exposure system for inhalation exposure studies
Source: Front Bioeng Biotechnol. 2026 Sep 1;14:1871869. doi: 10.3389/fbioe.2026.1871869 (PMC13574937; doi:10.3389/fbioe.2026.1871869)

Supplementary Information

Supplementary Information 1. Detailed valve logic and airflow pathways for Small-Loop and Large-Loop puff processes

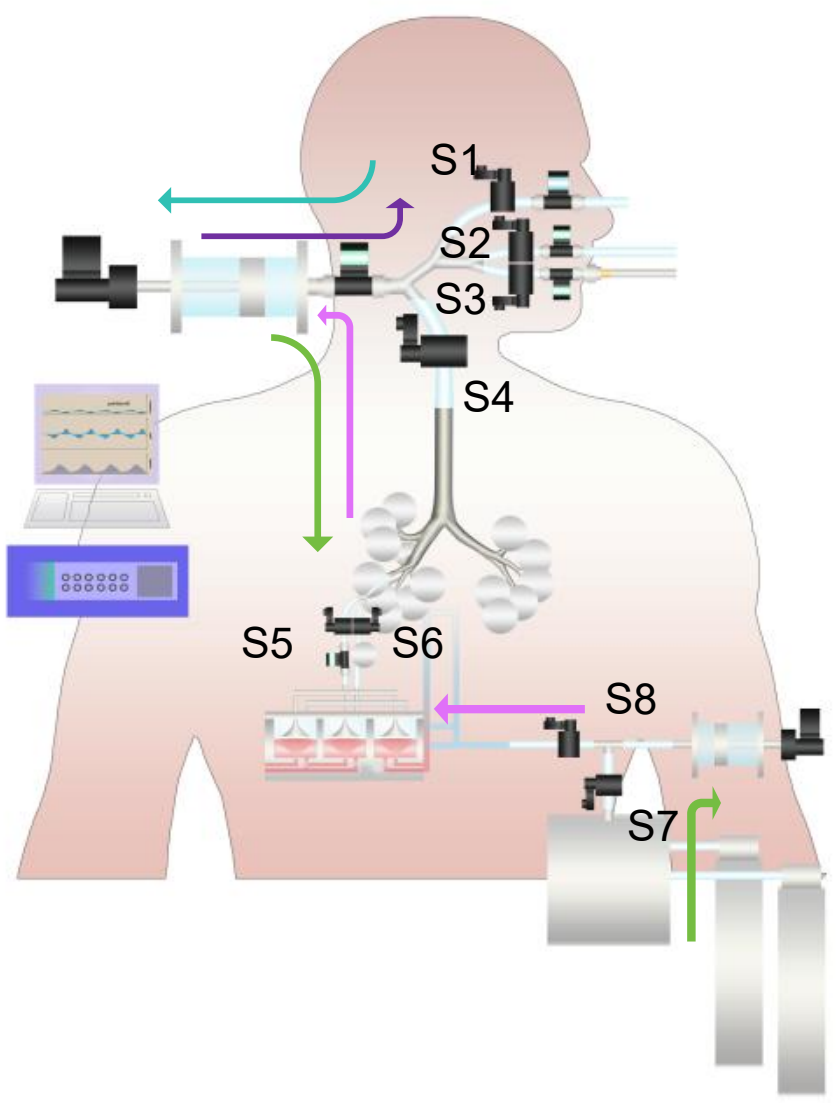

Small-Loop Puff Process

Phase I (Inhale)

✧ Valves: S1/S2/S3: Open (path-dependent); S4–S8: Closed

✧ Flow: Oral/Nasal → Pharynx → Syringe A

Breath-hold

✧ Valves: S1–S8: Closed

Phase II (Exhale):

✧ Valves: S1/S2/S3: Open (path-dependent); S4–S8: Closed

✧ Flow: Syringe A → Pharynx → Oral/Nasal

Large-Loop Puff Process

Phase I (Inhale I)

✧ Valves: S1/S2/S3: Open (path-dependent); S4–S8: Closed

✧ Flow: Oral/Nasal → Pharynx → Syringe A

Pause

✧ Valves: S1–S8: Closed

Phase II (Inhale II)

✧ Valves: S4, S5, S7: Open; S1-3, S6,S8: Closed

✧ Flow: Syringe A → Pharynx → Tracheobronchial tree → Lung exposure chambers ; CO_2 mixing chambers → Syringe B

Breath-hold

✧ Valves: S1–S8: Closed

Phase III (Exhale I)

✧ Valves: S8, S6, S4: Open; S1-3, S5,S7: Closed

✧ Flow: Syringe B → Lung exposure chambers → Tracheobronchial tree → Pharynx → Syringe A

Pause

✧ Valves: S1–S8: Closed

Phase IV (Exhale II)

✧ Valves: S1/S2/S3: Open (path-dependent); S4–S8: Closed

✧ Flow: Syringe A → Pharynx → Oral/Nasal

Supplementary Information 2. Schematic diagram of the programmable respiratory parameter control/monitoring system interface

(i) action setting interface

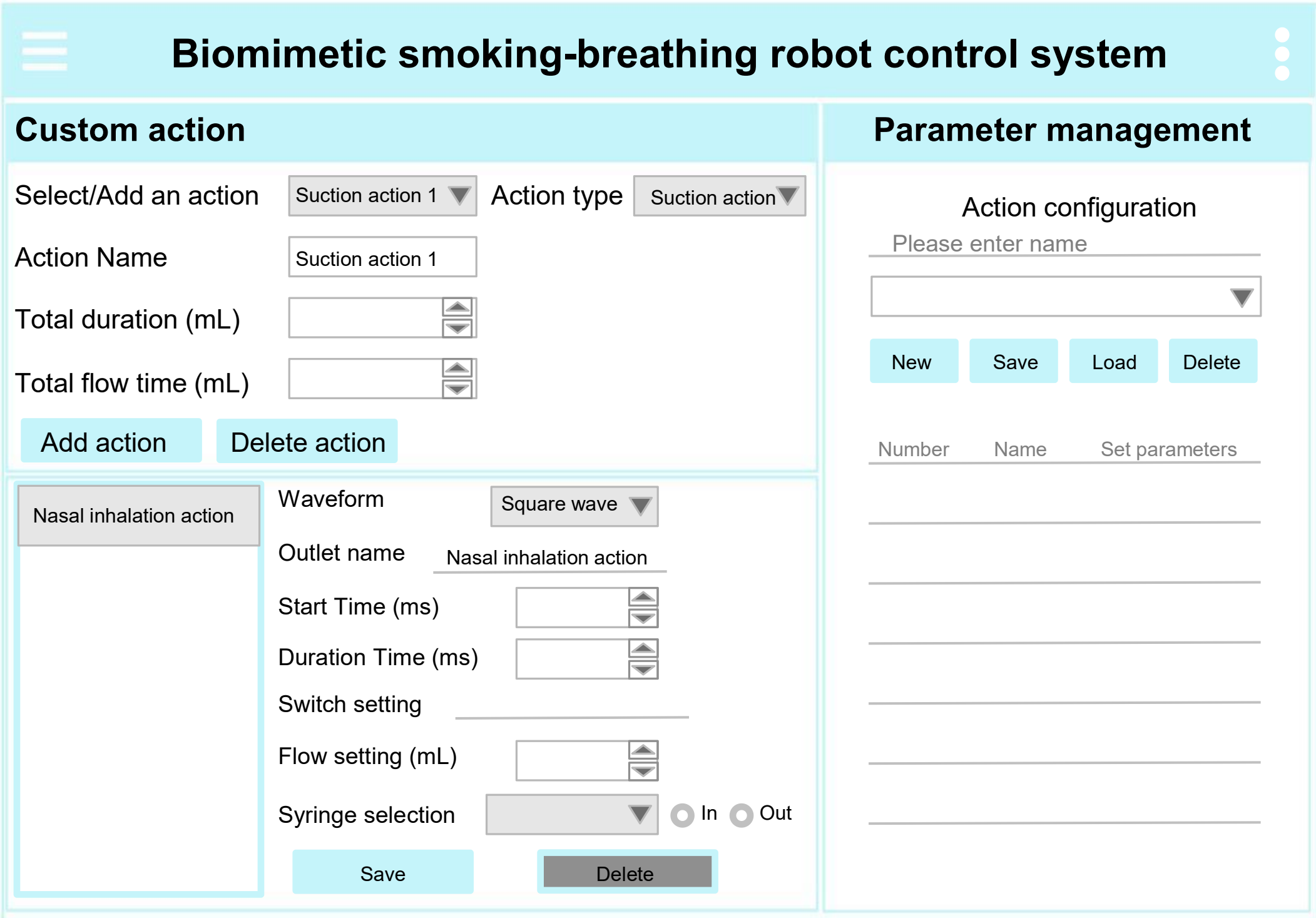

(ii) solenoid valve array control interface

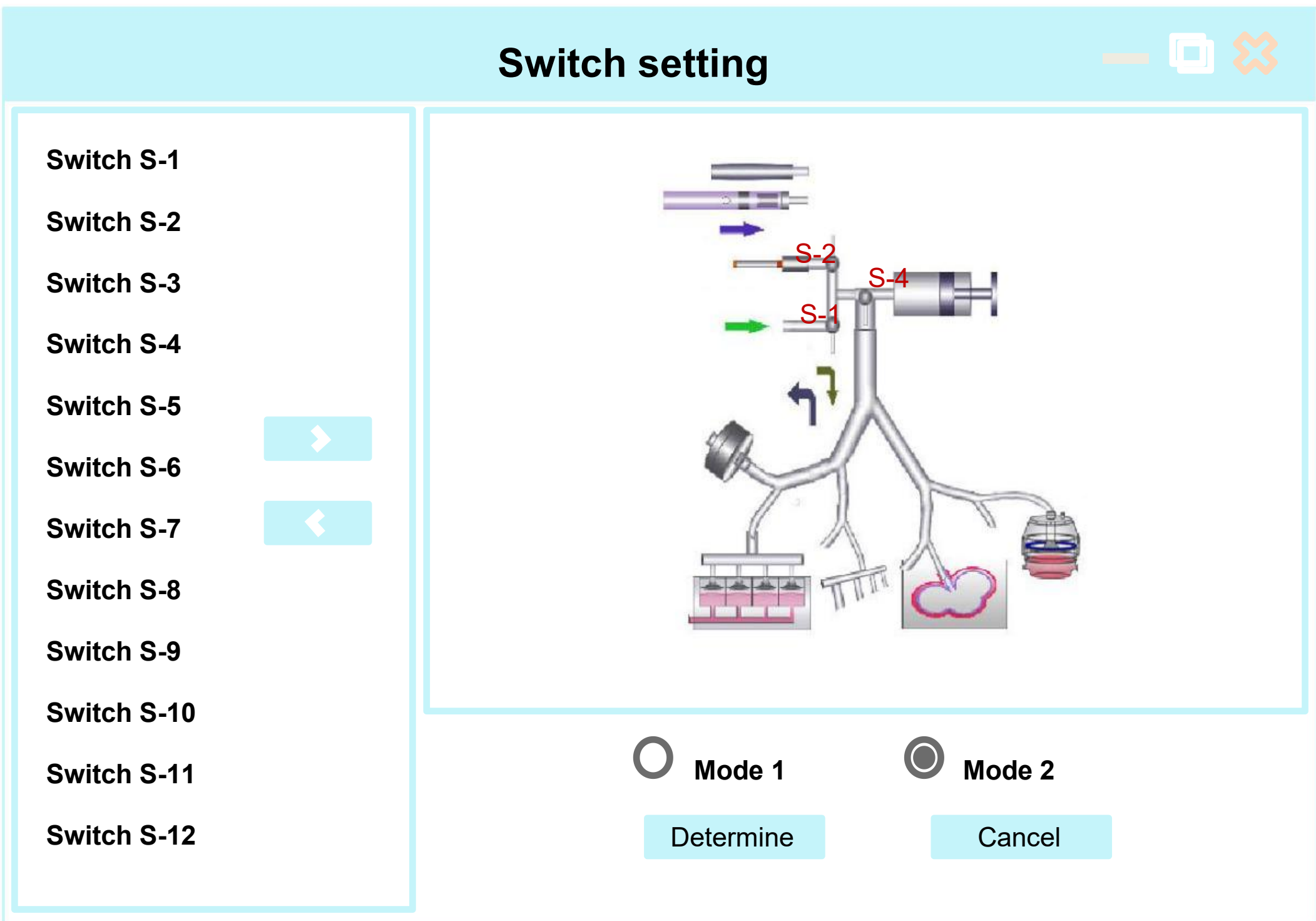

(iii) continuous action setting interface

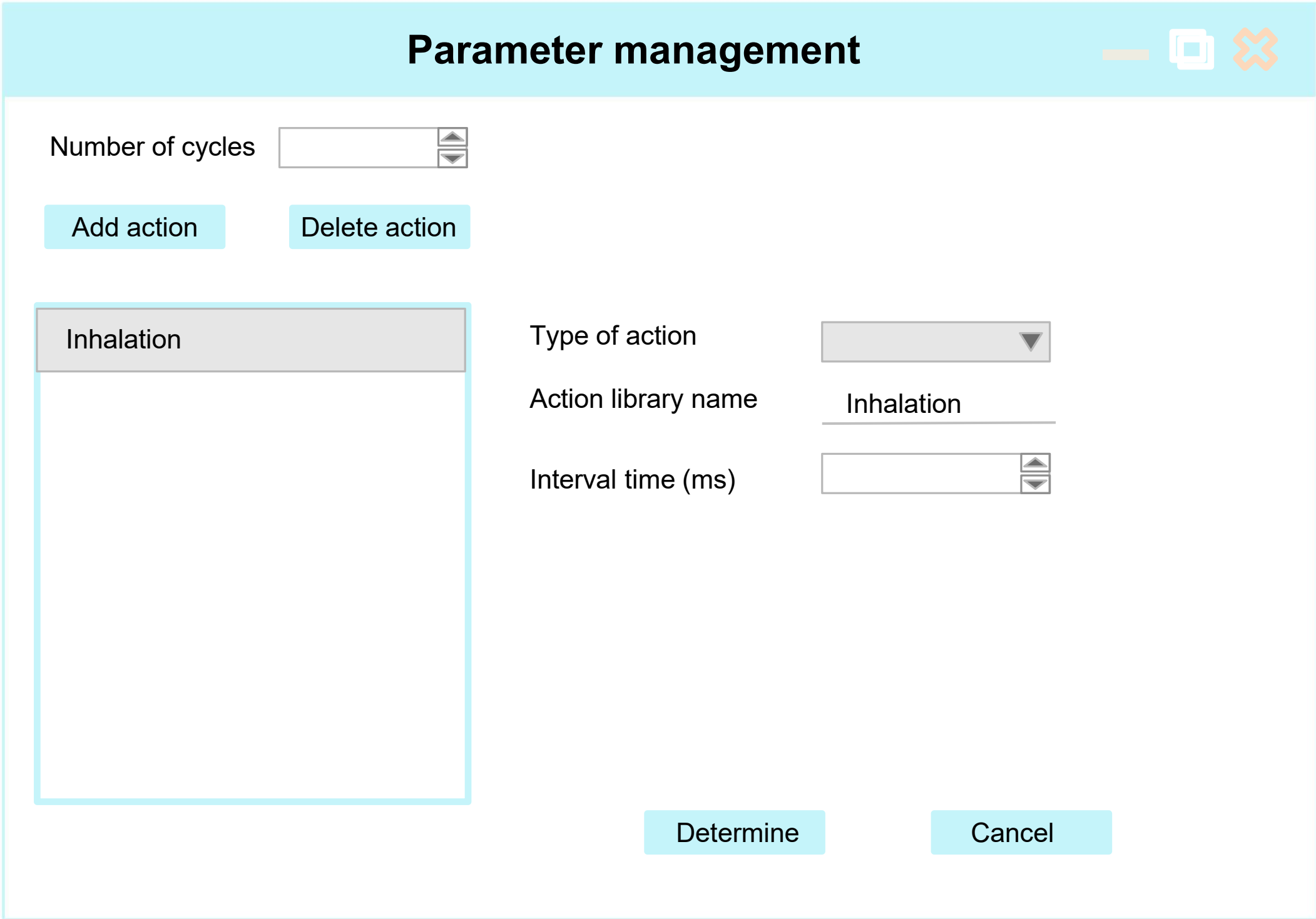

(iv) operation control/monitoring interface

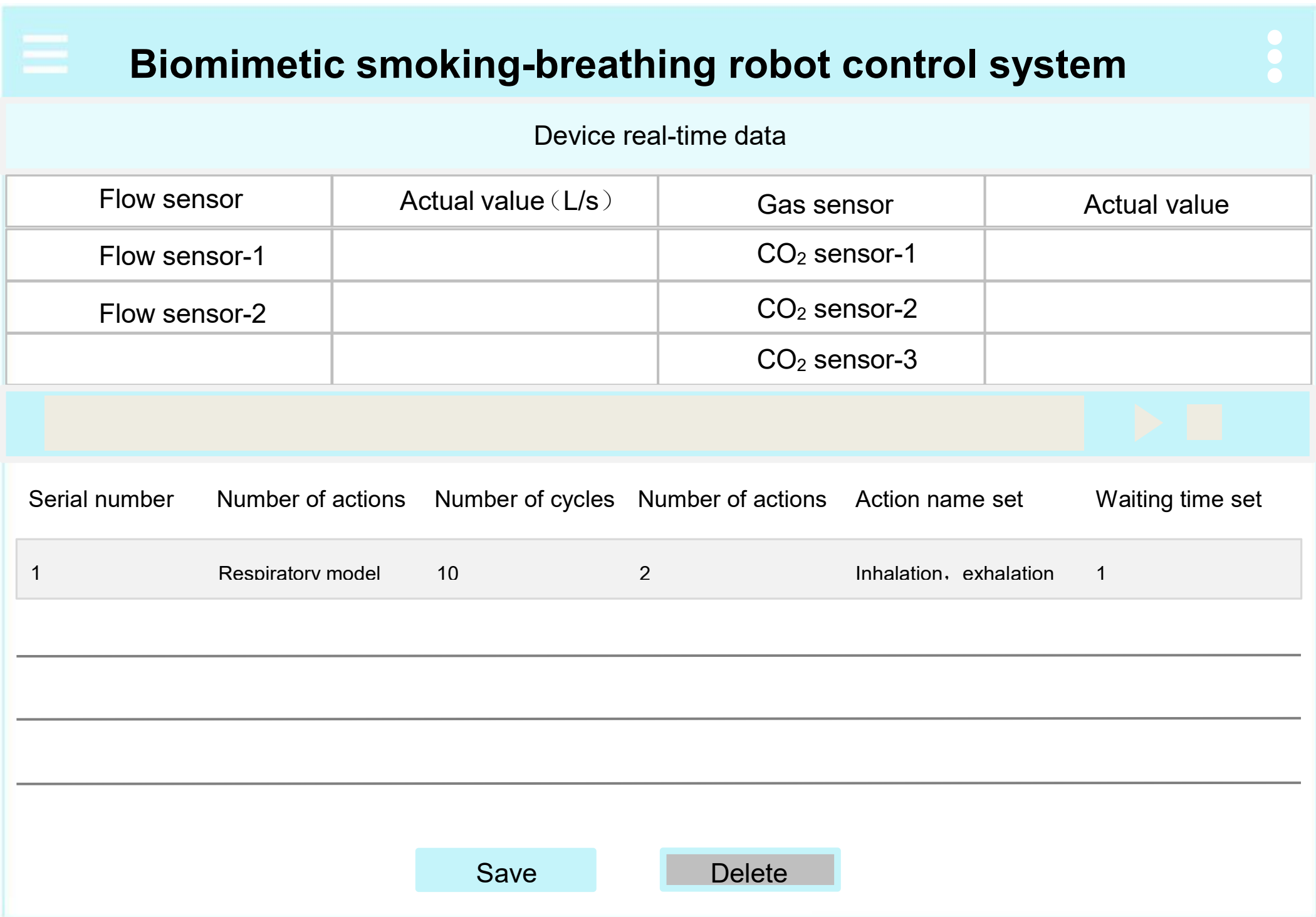

Supplementary Information 3. Control Algorithm Implementation Details

The control algorithm described in this note is implemented by the "Programmable Respiratory Parameter Control/Monitoring System (HZV1)". This system consists of four registered software copyright modules, which collectively realize the control logic of the following five functional modules: (1) System Configuration and Parameter Management (Inhalation-Exhalation Control Parameter Configuration Program V1.0, 2025SR0618862), (2) Waveform Generation (Bionic Respiratory Waveform Generation System V1.0, 2025SR1178324), (3) Volume and Time Control (Inhalation-Exhalation Control System V1.0, 2025SR0619028), (4) Action Definition → Action Combination → Program Execution, i.e., user-defined assembly and execution of respiratory cycles (Inhalation-Exhalation Control System V1.0, 2025SR0619028), and (5) CO_2 Feedback Control (CO_2 Concentration Control Software, 2025SR0457696).

1. System Configuration and Parameter Management

This section is implemented by the "Inhalation-Exhalation Control Parameter Configuration Program V1.0" (Software Copyright Registration No. 2025SR0618862). Before system operation, hardware parameters must be configured through this program:

- **Communication configuration:** Modbus TCP settings (IP address, port, polling interval) for PLC communication
- **Syringe configuration:** Syringe ID, volume capacity, timing parameters, waveform assignment, and Modbus communication addresses for command and status signals
- **Valve configuration:** Solenoid valve ID, type, on/off delay, module address, open/close commands, and status I/O
- **Sensor configuration:** Sensor ID, type, module address, data length, measurement range, and calibration factors

These parameters are saved and loaded by the main control program to ensure consistent hardware operation across different experimental setups.

2. Waveform Generation

This section is implemented by the "Bionic Respiratory Waveform Generation System V1.0" (Software Copyright Registration No. 2025SR1178324). This system implements five waveform formulas in the PLC program, defining how gas flow rate changes over time during inhalation or exhalation:

Waveform	Formula
Square wave	$V = V_0$
Triangular wave	$V = A(1 - \lvert 2t - T\rvert/T)$
Sine wave	$V = A(T - t)t$
Bell-shaped wave	$V = A\left[\sin\left(\frac{\pi t}{T}\right) + 2\right]$

Respiratory wave	$V = A\left[1 - \sin\left(\frac{\pi t}{2T}\right)\right]$

Key: V is the instantaneous velocity (i.e., the stepping pulse frequency at that moment, corresponding to instantaneous flow rate); t is the elapsed time; T is the total phase duration; A is automatically calculated by the PLC based on the user-set flow rate and total time; V_0 is the constant velocity.

The PLC calculates the instantaneous pulse frequency at each time step and sends corresponding pulses to the servo motor, driving the syringe to move according to the programmed flow-time profile.

3. Volume and Time Control

This section is implemented by the "Inhalation-Exhalation Control System V1.0" (Software Copyright Registration No. 2025SR0619028), which converts waveform commands into physical syringe motion:

- **Volume control:** After the user sets the target volume (mL), the software converts it into the required number of stepping pulses, precisely controlling syringe displacement and gas volume through the total pulse count
- **Time control:** The user sets the duration for each phase; the PLC's internal timers control the timing and trigger phase transitions when the programmed time is reached
- **Flow rate control:** Pulse frequency determines motor speed → syringe piston velocity → gas flow rate

Volume = Flow Rate × Time. The user sets any two parameters, and the system automatically calculates the third.

4. Action Definition → Action Combination → Program Execution

This section is implemented by the "Inhalation-Exhalation Control System V1.0" (Software Copyright Registration No. 2025SR0619028). The user defines and runs the complete respiratory cycle through three operational levels, and the system automatically repeats after completing each cycle.

4.1 Define Individual Actions

The split of inhalation volume between the oral and nasal routes is controlled by the flow diversion structures, which regulate the volume distribution by adjusting the cross-sectional area ratio of the oral and nasal path segments. By selecting different diversion structures, the user changes this area ratio and thereby sets the desired oral/nasal split. Examples are provided below.

In the "Custom Action" window (Supplementary Information 2 (i) action setting interface), the user sets parameters for a single action:

- Waveform (choose from five waveform types)
- Flow (mL)
- Time (ms)
- Syringe selection and mode (in and on)
- Solenoid valves (default closed; select to open; Supplementary Information 2 (ii) solenoid valve array control interface)
- Airflow path and corresponding valve and syringe configuration (refer to the table below)

The user adds each action with a custom name and saves the complete set of actions for

the same phase.
The valve and syringe configurations corresponding to each airflow path are as follows:

Airflow Path	Syringe	Mode	Valves Open	Valves Closed
Oral smoke → pharynx → syringe A	A	in (gas→syringe)	S3	S1, S2, S3–S8
Oral air → pharynx → syringe A	A	in (gas→syringe)	S2	S1, S3–S8
Nasal air → pharynx → syringe A	A	in (gas→syringe)	S1	S2–S8
Syringe A → pharynx → nasal exhaust	A	out (syringe→gas)	S1	S2–S8
Syringe A → pharynx → oral exhaust	A	out (syringe→gas)	S2	S1, S3–S8
Syringe A → pharynx → tracheobronchial tree → exposure chamber	A	out (syringe→gas)	S4, S5	S1–S3, S6–S8
CO_2 mixing chamber → syringe B	B	in (gas→syringe)	S7	S1–S5, S6, S8
Syringe B → exposure chamber	B	out (syringe→gas)	S8	S1–S7
Exposure chamber → tracheobronchial tree → pharynx → syringe A (withdrawal)	A	in (gas→syringe)	S4, S6	S1–S3, S5, S7–S8

Complete phase-by-phase valve configurations are provided in Supplementary Information 1 and 6.

Examples of Individual Actions

Example 1: Classic small-loop inhalation

- Path: Nasal (465 mL air) + Oral (35 mL smoke) → Pharynx → Syringe A (total 500 mL), 2 s, respiratory wave
- Select the corresponding flow diversion structure to achieve 465 mL nasal and 35 mL oral split
- Waveform: respiratory wave; Volume: 500 mL; Time: 2 s
- Valves open: S3 (oral), S1 (nasal)
- Syringe: A; Mode: in (gas→syringe)
- Name the action and save

Example 2: Classic small-loop exhalation

- Path: Syringe A → Pharynx → Nasal (500 mL), 2 s, respiratory wave
- Waveform: respiratory wave; Volume: 500 mL; Time: 2 s
- Valves open: S1 (nasal)
- Syringe: A; Mode: out (syringe→gas)
- Name the action and save

Example 3: Large-loop Phase II (dual-syringe simultaneous action)

Action A:

- Path: Syringe A → Pharynx → Tracheobronchial tree → lung exposure chambers (500 mL), 2 s
- Waveform: respiratory wave; Volume: 500 mL; Time: 2 s
- Valves open: S4, S5
- Syringe: A; Mode: out (syringe→gas)

Action B:

- Path: CO_2 mixing chambers → Syringe B (10 mL), 2 s
- Waveform: respiratory wave; Volume: 10 mL; Time: 2 s
- Valves open: S7

- Syringe: B; Mode: in (gas→syringe)

Combine Action A and Action B as a simultaneous action and save.

Example 4: Large-loop Phase III (dual-syringe simultaneous action)

Action A:

- Path: Syringe B → lung exposure chambers (CO_2 injection, 10 mL), 2 s
- Waveform: respiratory wave; Volume: 10 mL; Time: 2 s
- Valves open: S8
- Syringe: B; Mode: out (syringe→gas)

Action B:

- Path: lung exposure chambers → Syringe A (gas withdrawal, 510 mL), 2 s
- Waveform: respiratory wave; Volume: 510 mL; Time: 2 s
- Valves open: S4, S6
- Syringe: A; Mode: in (gas→syringe)

Combine Action A and Action B as a simultaneous action and save.

4.2 Combine Actions into a Program

In the "Action Configuration" window (Supplementary Information 2 (i) action setting interface and the "Continuous Action Setting" interface (Supplementary Information 2 (iii) continuous action setting interface), the user concatenates multiple actions into a complete program:

- Name the combined action in the "Action Configuration" window
- Enter the "Continuous Action Setting" interface and set the number of cycles
- Call previously saved individual actions and set the interval time to the next action (breath-hold or pause)
- Add actions one by one until all actions for one cycle are added

4.3 Execute the Program

In the "Program List" interface (Supplementary Information 2 (iv) operation control/monitoring interface), the user selects a saved program and clicks "Execute". The system runs through the defined sequence step by step, displays progress in the progress bar, shows real-time sensor data, and can be paused or stopped at any time.

5. CO_2 Feedback Control

This section is implemented by the "CO_2 Concentration Control Software" (Software Copyright Registration No. 2025SR0457696), which runs in parallel with the breathing program.

5.1 Feedback Logic

The CO_2 sensor continuously measures the concentration in the exposure chamber. The software compares the measured value with the user-set target and controls the CO_2 and N_2 injection valves accordingly:

Condition	CO_2 Valve	N_2 Valve
Measured < Target − Deviation	Open	Closed
Measured > Target + Deviation	Closed	Open
Target − Deviation ≤ Measured ≤ Target + Deviation	Closed	Closed

5.2 Deviation Value Settings

To prevent rapid valve switching, the software provides adjustable deviation values. The user can configure through the software interface:

- **Valve-open deviation:** Opens the CO_2 valve when the concentration falls below "target − deviation"
- **Valve-close deviation:** Closes the CO_2 valve when the concentration exceeds "target + deviation"
- **N_2 upper/lower limits:** Define the operating range for the N_2 valve

When the concentration is within the range of "target ± deviation", both valves remain closed, and the CO_2 concentration remains stable.

5.3 Integration with the Breathing Program

When CO_2 is injected during the exhalation phase of the breathing program, the CO_2 feedback control module ensures that the concentration in the exposure chamber stabilizes at the target level. The feedback loop continuously compensates for any drift to maintain the setpoint.

Summary

The above four software copyrights have been officially registered with the National Copyright Administration of China. The public can query basic registration information by entering the registration numbers in the "Registration Announcement" section of the official website (www.ccopyright.com.cn). This supplementary Information, together with the main text and the referenced software copyrights, constitutes a complete description of the control algorithm.

Supplementary Information 4. Technical specifications of the bionic respiratory airway exposure system

- ✓ Respiratory Volume Parameters
  - ✧ Tidal volume (inhalation/exhalation): 0–1000 mL
  - ✧ Special inhalation capacity: Adjustable within tidal volume range
- ✓ Respiratory Timing Parameters
  - ✧ Inhalation/exhalation duration: 0–60 sec
  - ✧ Special inhalation duration: Programmable within cycle
  - ✧ Respiratory frequency: 0–20 breaths/min
- ✓ Interval Parameters
  - ✧ Breath-holding intervals:
    - ➢ Post-inspiratory pause (0–10 min)
    - ➢ Post-expiratory pause (0–10 min)
    - ➢ Special inhalation interval (0–10 min)
- ✓ Flow Waveform Options
  - ✧ Standard breathing waveforms:
    - ➢ Breathing
    - ➢ Sinusoidal
    - ➢ Bell-shaped
    - ➢ Triangular
    - ➢ Square
- ✓ Gas Composition Control
  - ✧ CO_2 mixing chamber output concentrations: 0–100%
  - ✧ Real-time gas concentration monitoring
    - ➢ CO_2: 0–100% (physiological range 3–6%)
    - ➢ O_2: 0–100% (physiological range 16–21%)

Supplementary Information 5. Parameters for smoking and breathing processes in three scenarios

- ✓ Small-Loop Puff Process
  - ✧ Phase I: Syringe A inhales through the oral + nasal (tidal volume: 500 mL, inhalation duration: 2 s; waveform simulates natural breathing, resembling a sine wave—flow rate rapidly peaks and gradually declines to zero). It draws two gas streams into Syringe A:
    - ➢ Oral: Smoked gas (35 mL)
    - ➢ Nasal: Air (465 mL)
  - ❖ After completion, breath-hold for 1 s.
  - ✧ Phase II: Syringe A exhales the gas (tidal volume: 500 mL, exhalation duration: 2 s, respiratory waveform) through the nasal (500 mL).
  - ❖ Then breath-holds for 1 s before the next process.
- ✓ Tidal Breath Process
  - ✧ Phase I: Syringe A inhales through the nasal (tidal volume: 500 mL, inhalation duration: 2 s, respiratory waveform), drawing mixed gas into Syringe A.
  - ❖ Pause for 0.2 s after completion.

- Phase II: Syringe A pushes the gas (500 mL, inhalation duration: 2 s, respiratory waveform) into the trachea → pulmonary modules + exposure chamber (21.53 ± 0.24 mL, 0.04% CO_2). Simultaneously, Syringe B draws 16% CO_2 (10 mL, duration: 2 s, respiratory waveform).
- Breath-hold for 1 s.
- Phase III: Syringe B pushes the 16% CO_2 (10 mL, duration: 2 s, respiratory waveform) into the liquid line of the exposure chamber, where CO_2 diffuses into the liquid layer and then the gas layer (final concentration in exposure chamber: 3.57% CO_2, normal exhaled CO_2 level). Meanwhile, Syringe A draws gas from the exposure chamber and pulmonary modules (including CO_2 from the gas layer) back into itself.
- Pause for 0.2 s.
- Phase IV: Syringe A exhales the gas (tidal volume: 510 mL, exhalation duration: 2 s, respiratory waveform) through the oral.
- Then breath-holds for 1 s before the next process.

✓ Large-Loop Puff Process

- Phase I: Syringe A inhales through the oral + nasal (tidal volume: 500 mL, inhalation duration: 2 s, respiratory waveform), drawing:
  - Oral: Smoked gas (35 mL)
  - Nasal: Air (465 mL)
- Pause for 0.2 s after completion.
- Phases II & III: Same as Tidal Breath Process (Phases II & III).
- Phase IV: Syringe A exhales the gas (tidal volume: 510 mL, exhalation duration: 2 s, respiratory waveform) through the nasal.
- Then breath-holds for 1 s before the next process.

✓ Customized Small-Loop Puff Process

- Phase I: Syringe A inhales through the oral (smoke volume: 300 mL, inhalation duration: 1.5 s, sine wave, typical for ventilators), drawing smoke into Syringe A.
- Breath-hold for 2 s.
- Phase II: Syringe A exhales the smoke (tidal volume: 300 mL, exhalation duration: 3 s, square wave, as in volume-controlled mechanical ventilation) through oral + nasal.
- Then breath-holds for 2 s before the next process.

✓ Customized Large-Loop Puff Process

- Phase I: Syringe A inhales through the oral + nasal (tidal volume: 500 mL, inhalation duration: 2 s, respiratory waveform), drawing:
  - Oral: Smoked gas (55 mL)
  - Nasal: Air (445 mL)
- Pause for 0.2 s after completion.
- Phases II & III: Similar to Breathing Process (Phases II & III), but using a square wave.
- Phase IV: Syringe A exhales the gas (tidal volume: 510 mL, exhalation duration: 2 s, respiratory waveform) through the nasal.
- Then breath-holds for 1 s before the next process.

- ✓ Personalized Breathing Process
  - ✧ Phase I: Syringe A inhales through the oral + nasal (tidal volume: 800 mL, inhalation duration: 3 s, bell-shaped wave, recommended for cigarette smoking), drawing mixed gas into Syringe A.
  - ❖ Pause for 0.2 s.
  - ✧ Phase II: Syringe A pushes the gas (800 mL, inhalation duration: 3 s, sine wave) into the trachea → pulmonary modules + exposure chamber (33.25 ± 0.16 mL, 0.04% CO_2). Simultaneously, Syringe B draws 30% CO_2 (30 mL, duration: 2 s, sine wave).
  - ❖ Breath-hold for 1 s.
  - ✧ Phase III: Syringe B pushes the 30% CO_2 (10 mL, duration: 2 s, triangular wave) into the exposure chamber (pathological exhaled CO_2 concentration). Meanwhile, Syringe A draws gas from the exposure chamber and pulmonary modules (830 mL, duration: 3 s, triangular wave, simulating COPD patient ventilation—flow rate rises to peak and then declines).
  - ❖ Pause for 0.2 s.
  - ✧ Phase IV: Syringe A exhales the gas (tidal volume: 830 mL, exhalation duration: 3 s, respiratory waveform) through the oral.
  - ❖ Then breath-holds for 3 s before the next process.

Supplementary Information 6. Phase-by-phase breakdown of breathing patterns

This table provides a complete phase-by-phase description of each breathing pattern, including the breathing inlet/outlet, tidal volume, waveform, duration, CO_2-related parameters, valve configurations (S1–S8), airflow route, and control type for each phase (I–IV). "–" indicates not applicable.

Breathing Mode	Phase	Breathing Inlet / Outlet	Tidal Volume (mL)	Waveform	Duration (s)	CO_2-Related Parameters	Valves	Route	Control Type
Small-loop puff	Phase I (Inhale)	Nasal + Oral (Nasal: 465 air, Oral: 35 smoke)	500	Sine	2	None	Open: S1, S2<br>Closed: S3- S8	Nasal + Oral → Pharynx → Syringe A	Open-loop
	Breath-hold	–	–	–	1	–	Closed: all	–	–
	Phase II (Exhale)	Nasal	500	Respiratory	2	None	Open: S1<br>Closed: S2- S8	Syringe A → Pharynx → Nasal	Open-loop
	Breath-hold	–	–	–	1	–	Closed: all	–	–
Tidal breath	Phase I (Inhale I)	Nasal	500	Respiratory	2	None	Open: S1<br>Closed: S2- S8	Nasal → Pharynx → Syringe A	Closed-loop (CO_2 feedback)
	Pause	–	–	–	0.2	–	Closed: all	–	–
	Phase II (Inhale II)	–	500	Respiratory	2	Syringe B draws 16% CO_2 10 mL	Open: S4, S5, S7<br>Closed: S1- S3, S6, S8	Syringe A → Pharynx → Tracheobronchial tree → lung exposure chambers;<br>CO_2 mixing chambers → Syringe B	Closed-loop
	Breath-hold	–	–	–	1	–	Closed: all	–	–
	Phase III (Exhale I)	–	–	Respiratory	2	CO_2 diffuses → 3.57% in chamber	Open: S4, S6, S8;<br>Closed: S1- S3, S5, S7	Syringe B → lung exposure chambers → Tracheobronchial tree → Pharynx → Syringe A	Closed-loop
	Pause	–	–	–	0.2	–	Closed: all	–	–
	Phase IV (Exhale II)	Oral	510	Respiratory	2	–	Open: S3<br>Closed: S1, S2, S4- S8	Syringe A → Pharynx → Oral	Closed-loop
	Breath-hold	–	–	–	1	–	Closed: all	–	–
Large-loop puff	Phase I (Inhale I)	Nasal + Oral (Nasal: 465 air, Oral: 35 smoke)	500	Respiratory	2	None	Open: S1, S2<br>Closed: S3- S8	Nasal + Oral → Pharynx → Syringe A	Closed-loop
	Pause	–	–	–	0.2	–	Closed: all	–	–

	Phase II (Inhale II)	–	500	Respiratory	2	16% CO_2 10 mL drawn	Open: S4, S5, S7 Closed: S1- S3, S6, S8	Syringe A → Pharynx → Tracheobronchial tree → lung exposure chambers; CO_2 mixing chambers → Syringe B	Closed-loop
	Breath-hold	–	–	–	1	–	Closed: all	–	–
	Phase III (Exhale I)	–	–	Respiratory	2	→ 3.57% CO_2 in chamber	Open: S4, S6, S8 Closed: S1- S3, S5, S7	Syringe B → lung exposure chambers → Tracheobronchial tree → Pharynx → Syringe A	Closed-loop
	Pause	–	–	–	0.2	–	Closed: all	–	–
	Phase IV (Exhale II)	Nasal	510	Respiratory	2	–	Open: S1 Closed: S2- S8	Syringe A → Pharynx → Nasal	Closed-loop
	Breath-hold	–	–	–	1	–	Closed: all	–	–
Customized small-loop puff	Phase I (Inhale)	Oral	300	Sine	1.5	None	Open: S2 Closed: S1, S3- S8	Oral → Pharynx → Syringe A	Open-loop
	Breath-hold	–	–	–	2	–	Closed: all	–	–
	Phase II (Exhale)	Nasal + Oral	300	Square	3	None	Open: S1, S3 Closed: S2, S4- S8	Syringe A → Pharynx → Nasal + Oral	Open-loop
	Breath-hold	–	–	–	2	–	Closed: all	–	–
Customized large-loop puff	Phase I (Inhale I)	Nasal + Oral (Nasal: 445 air, Oral: 55 smoke)	500	Respiratory	2	None	Open: S1, S2 Closed: S3- S8	Nasal + Oral → Pharynx → Syringe A	Closed-loop
	Pause	–	–	–	0.2	–	Closed: all	–	–
	Phase II (Inhale II)	–	500	Square	2	16% CO_2 10 mL drawn	Open: S4, S5, S7 Closed: S1- S3, S6, S8	Syringe A → Pharynx → Tracheobronchial tree → lung exposure chambers; CO_2 mixing chambers → Syringe B	Closed-loop
	Breath-hold	–	–	–	1	–	Closed: all	–	–
	Phase III (Exhale I)	–	–	Square	2	→ 3.57% CO_2 in chamber	Open: S4, S6, S8 Closed: S1- S3, S5, S7	Syringe B → lung exposure chambers → Tracheobronchial tree → Pharynx → Syringe A	Closed-loop
	Pause	–	–	–	0.2	–	Closed: all	–	–
	Phase IV (Exhale II)	Nasal	510	Respiratory	2	–	Open: S1 Closed: S2- S8	Syringe A → Pharynx → Nasal	Closed-loop

	Breath-hold	–	–	–	1	–	Closed: all	–	–
Personalized breathing	Phase I (Inhale I)	Nasal + Oral	800	Bell	3	None	Open: S1, S3<br>Closed: S2, S4- S8	Nasal + Oral → Pharynx → Syringe A	Closed-loop (CO_2 feedback + pathological)
	Pause	–	–	–	0.2	–	Closed: all	–	–
	Phase II (Inhale II)	–	800	Sine	3	Syringe B draws 30% CO_2 10 mL	Open: S4, S5, S7<br>Closed: S1- S3, S6, S8	Syringe A → Pharynx → Tracheobronchial tree → lung exposure chambers; CO_2 mixing chambers → Syringe B	Closed-loop
	Breath-hold	–	–	–	1	–	Closed: all	–	–
	Phase III (Exhale I)	–	830 (withdrawn)	Triangular (withdraw) / Sine (inject)	2 (inject) / 3 (withdraw)	30% CO_2 → pathological CO_2	Open: S4, S6, S8<br>Closed: S1- S3, S5, S7	Syringe B → lung exposure chambers → Tracheobronchial tree → Pharynx → Syringe A	Closed-loop
	Pause	–	–	–	0.2	–	Closed: all	–	–
	Phase IV (Exhale II)	Oral	830	Respiratory	3	–	Open: S3<br>Closed: S1, S2, S4- S8	Syringe A → Pharynx → Oral	Closed-loop
	Breath-hold	–	–	–	3	–	Closed: all	–	–

Supplementary Information 7. Reagents related to hiPSC-derived lung model differentiation

Table S1: Reagents for human induced pluripotent stem cells (hiPSCs) culture.

Table S1. Reagents for hiPSCs culture

Compound	Company	Cat#	Stock [c]	Final [c]
Y-27632	Sigma	SCM075	10mM	10μM
mTeSR™ medium	STEMCELL	85851	-	-
Matrigel	Corning	354277	-	-
Accutase	Gibco	A1110501	-	-
Trypan Blue	Gibco	15250061	-	-
DPBS	Gibco	14190-144	-	-

Table S2: Composition of the basal differentiation medium.

Table S2. Composition of the basal differentiation medium

Compound	Company	Cat#	Stock [c]	Final [c]
DMEM/F-12, GlutaMAX	Gibco	10565018	-	-
B-27 (50X)	Gibco	17504044	50 X	1 X
N-2 (100X)	Gibco	17502048	100 X	1 X
penicillin-streptomycin	Gibco	15140122	10,000 U/mL	50 U/mL
fetal bovine serum	Gibco	10100147	-	2%
L-Ascorbic acid	R&D systems	4055/50	100 mg/mL	50 μg/mL
Monothioglycerol	Sigma-Aldrich	M6145	0.15 mol/mL	400 μM

Table S3: Factors for definitive endoderm (DE) differentiation.

Table S3. Factors for DE differentiation

Compound	Company	Cat#	Stock[c]	Final [c]	Time
rhBMP4	R&D systems	314-BPE-010	100 μg/mL	3 ng/mL	day 1
Y-27632	Sigma	SCM075	10 mM	10 μM	day 1,2
Activin A	R&D systems	338-AC-050	100 μg/mL	100 ng/mL	day 2-4
CHIR99021	Selleck	S2924	5 mM	1 μM	day 2-4

Table S4: Factors for anterior foregut endoderm (AFE) differentiation.

Table S4. Factors for AFE differentiation

Compound	Company	Cat#	Stock [c]	Final [c]	Time
SB431542	Tocris bioscience	1614	50 mM	10 μM	day 5-7
Noggin	R&D systems	6057-NG-100	250 μg/mL	100 ng/mL	

Table S5: Factors for lung progenitor (LPA) differentiation.

Table S5. Factors for LPA differentiation

Compound	Company	Cat#	Stock [c]	Final [c]	Time
CHIR99021	Selleck	S2924	5 mM	3 μM	day 8-17

rhBMP-4 (E. coli)	R&D systems	314-BPE-010	100 µg/mL	10 ng/mL	
all trans retinoic acid (ATRA)	Sigma-Aldrich	R2625	1 mM	1 µM	
rhKGF/FGF-7	R&D systems	251-KG-010	100 µg/mL	10 ng/mL	
rhFGF-10	R&D systems	345-FG-025	100 µg/mL	10 ng/mL	
rhEGF	R&D systems	236-EG-200	100 µg/mL	20 ng/mL	

Table S6: Factors for human induced pluripotent stem cell-derived lung model differentiation.

Table S6. Factors for hiPSC-lung model differentiation

Compound	Company	Cat#	Stock [c]	Final [c]	Time
CHIR99021	Selleck	S2924	5 mM	3 µM	day 18-27
rhKGF/FGF-7	R&D systems	251-KG-010	100 µg/mL	10 ng/mL	
rhFGF-10	R&D systems	345-FG-025	100 µg/mL	10 ng/mL	
Dexamethasone	Sigma-Aldrich	D4902-100MG	0.1 mM	50 nM	
8-Br-cAmp	Sigma-Aldrich	B5386	0.1 M	0.1 mM	
IBMX	Sigma-Aldrich	I5879	0.5 M	0.1 mM	

Supplementary Table S7: Antibodies used for immunofluorescence staining.

(KRT5, ACTUB, CC10, MUC5AC), distal alveolar epithelial lineage markers (PDPN, SP-C, SP-B), and markers for other cell populations (CHGA, SOX2, SOX9, PDGFR-α, CD31, CD68).

Table S7. Antibodies used for immunofluorescence staining

Compound	Company	Cat#	Dilution
Anti-SOX2	Abcam	ab92494	1:100
Anti-MUC5AC	Cell Signaling Technology	61193	1:400
Anti-Keratin5 (KRT5)	Cell Signaling Technology	71536	1:400
Podoplanin (PDPN)	Biolegend	337002	1:50
Anti-Pro-SPB	Abcam	ab271345	1:500
Anti-CD68	Cell Signaling Technology	76437	1:400
Anti-CD31	Cell Signaling Technology	3528	1:800
Anti-SOX9	Abcam	Ab185966	1:200
Anti-PDGF Receptor alpha α (PDGF-α)	Cell Signaling Technology	3174S	1:200
Anti-EPCAM	Cell Signaling Technology	2929	1:800
Acetyl-α-Tubulin (AcTub)	Cell Signaling Technology	5335	1:800

Anti-Uteroglobin (CC10)	Abcam	ab307666	1:1000
Anti-CHGA	Abcam	Ab283265	1:50
Anti-Pro-SPC	Abcam	ab90716	1:1000
Goat Anti-Rabbit IgG H&L488	Abcam	ab150077	1:500

Supplementary Information 8. Time-flow waveforms under threethree characteristic inhalation-smoking patterns

(a) Time-flow waveforms at multiple monitoring sites during classic small-loop smoking with breathing pattern

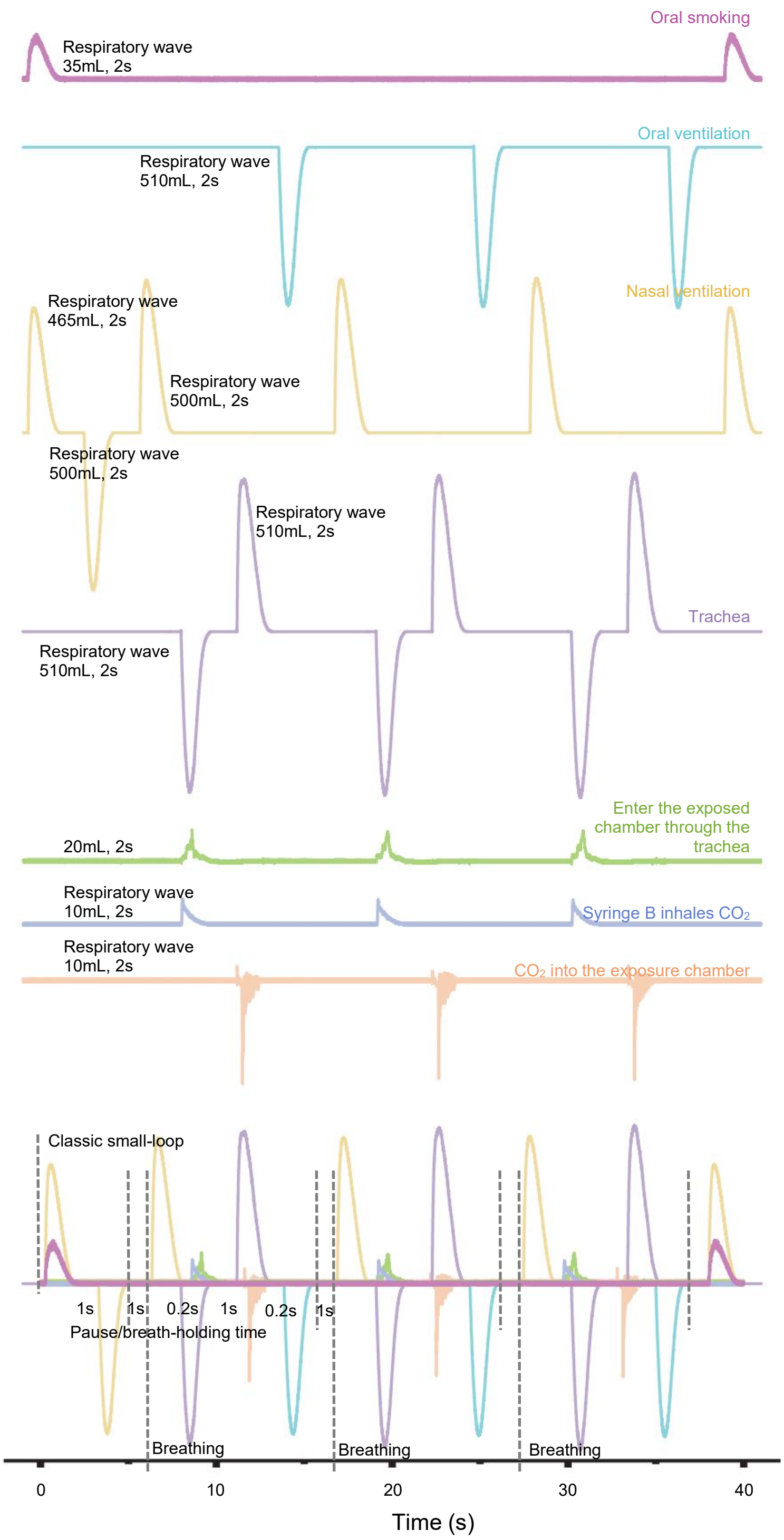

(b) Time-flow waveforms at multiple monitoring sites during classic large-loop smoking with breathing pattern

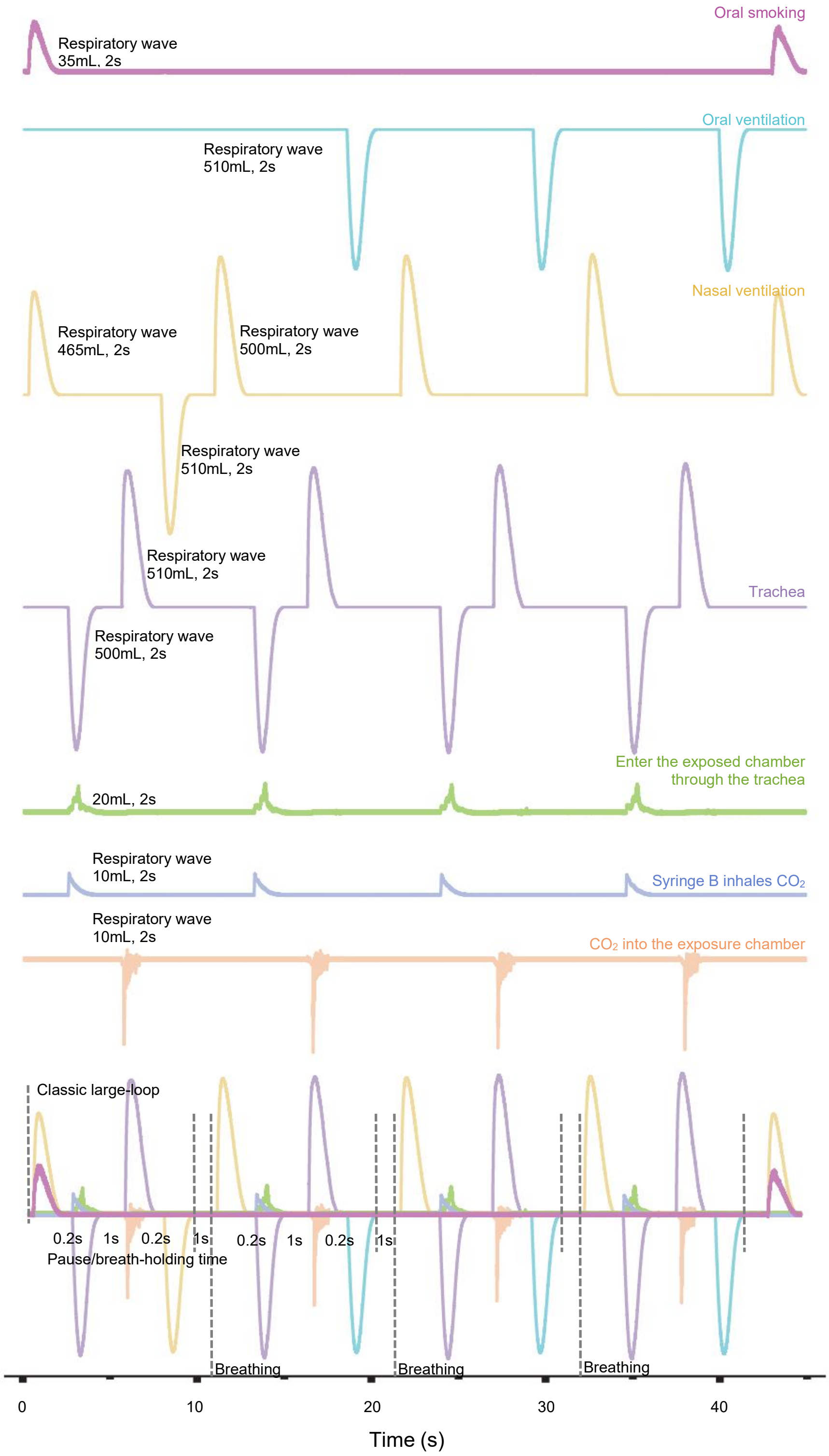

(c) Time-flow waveforms at multiple monitoring sites during personalized smoking with breathing pattern

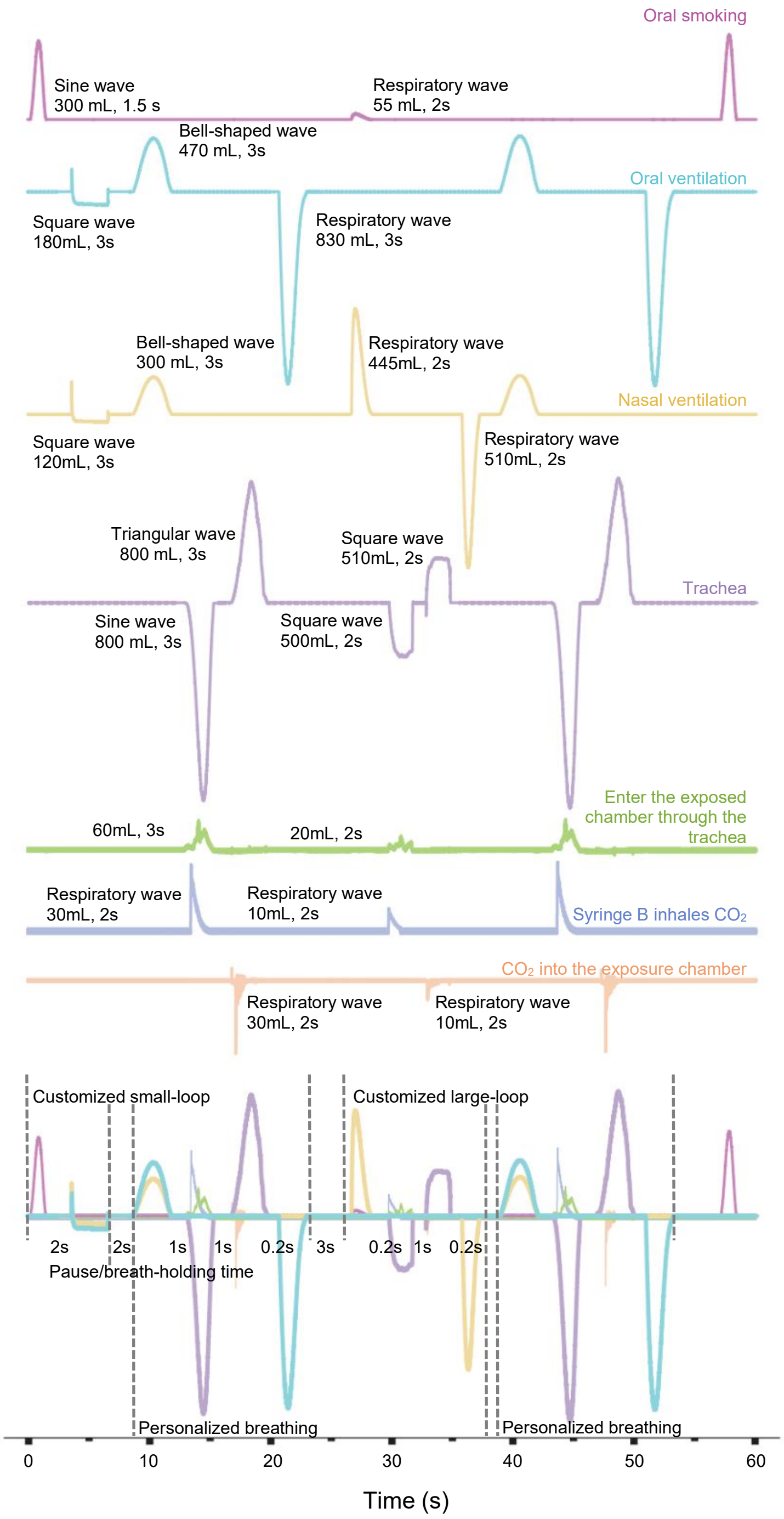

Supplement: Supplementary file 1 [file DataSheet1.pdf]
